# Progesterone, via yes‐associated protein, promotes cardiomyocyte proliferation and cardiac repair

**DOI:** 10.1111/cpr.12910

**Published:** 2020-10-12

**Authors:** Cong Lan, Nian Cao, Caiyu Chen, Shuang Qu, Chao Fan, Hao Luo, Andi Zeng, Cheng Yu, Yuanzheng Xue, Hongmei Ren, Liangpeng Li, Hongyong Wang, Pedro A. Jose, Zaicheng Xu, Chunyu Zeng

**Affiliations:** ^1^ Department of Cardiology Daping Hospital The Third Military Medical University Chongqing China; ^2^ Chongqing Institute of Cardiology Chongqing China; ^3^ Division of Renal Diseases & Hypertension Departments of Medicine and Pharmacology/Physiology The George Washington University School of Medicine and Health Sciences Washington DC USA; ^4^ Cardiovascular Research Center Chongqing College University of Chinese Academy of Sciences Chongqing China

## Abstract

**Objectives:**

The mechanisms responsible for the postnatal loss of mammalian cardiac regenerative capacity are not fully elucidated. The aim of the present study is to investigate the role of progesterone in cardiac regeneration and explore underlying mechanism.

**Materials and Methods:**

Effect of progesterone on cardiomyocyte proliferation was analysed by immunofluorescent staining. RNA sequencing was performed to screen key target genes of progesterone, and yes‐associated protein (YAP) was knocked down to demonstrate its role in pro‐proliferative effect of progesterone. Effect of progesterone on activity of YAP promoter was measured by luciferase assay and interaction between progesterone receptor and YAP promoter by electrophoretic mobility shift assay (EMSA) and chromatin immunoprecipitation (ChIP). Adult mice were subjected to myocardial infarction, and then, effects of progesterone on adult cardiac regeneration were analysed.

**Results:**

Progesterone supplementation enhanced cardiomyocyte proliferation in a progesterone receptor‐dependent manner. Progesterone up‐regulated YAP expression and knockdown of YAP by small interfering RNA reduced progesterone‐mediated cardiomyocyte proliferative effect. Progesterone receptor interacted with the YAP promoter, determined by ChIP and EMSA; progesterone increased luciferase activity of YAP promoter and up‐regulated YAP target genes. Progesterone administration also promoted adult cardiomyocyte proliferation and improved cardiac function in myocardial infarction.

**Conclusion:**

Our data uncover a role of circulating progesterone withdrawal as a novel mechanism for the postnatal loss of mammalian cardiac regenerative potential. Progesterone promotes both neonatal and adult cardiomyocyte proliferation by up‐regulating YAP expression.

## Graphical abstract

Progesterone promotes cardiomyocyte proliferation by transcriptional activation of YAP.

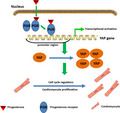

## 1 INTRODUCTION

Heart failure, caused by ischemic heart disease, as in myocardial infarction (MI), is one of the major causes of mortality worldwide.^1^ Despite remarkable advances in the development of pharmacological and technological treatments for MI, irreversible loss of cardiomyocytes (CMs) makes the incidence of heart failure remain high.^2^ Thus, intensive research has focused on the development of regenerative therapies to replace lost myocardium.^3^

One paradigm‐breaking concept has recently emerged that the adult mammalian CM is still endowed with proliferative potential, although the capacity is insufficient to replenish lost CMs.^4^ In particular, a ^14^C dating study showed an annual CM renewal rate of 0.45%‐1% in adult humans.^5^ Therefore, regenerating CMs in situ, via stimulation of endogenous CM proliferation, is an appealing approach to treat myocardial diseases associated with cell death, for example, MI. Large amount of evidences have demonstrated that foetal and neonatal CMs possess robust proliferative capacity and injured CMs during this period can be fully replaced by newly generated CMs.^6^, ^7^ However, this regenerative capacity quickly diminishes by postnatal day 7 (P7) in mice. Thus, discovery of regulators that account for the withdrawal of CM’s regenerative property after birth could help uncover new approaches to induce cell cycle re‐entry in adult CMs.

Circulatory hormones, especially progesterone, change drastically after birth.^8^ During foetal development, when CMs are rapidly proliferating, the foetus is supplied with progesterone produced by the placenta and/or the ovarian corpus luteum.^9^ Increased circulating level of progesterone plays a crucial role in the maintenance of pregnancy through inhibition of oxytocin‐induced myometrial activity.^10^ Notably, progesterone is also important in the regulation of foetal development and growth.^11^ However, the relationship between the decrease in foetal progesterone level and the postnatal withdrawal of the CM’s regenerative property is unknown. In the current study, we first show that serum progesterone level drastically declines from foetal to neonatal mice, which is in parallel with the withdrawal of cardiac regenerative capacity. Furthermore, progesterone supplementation, by up‐regulating Yes‐associated protein (YAP) expression, promotes postnatal CM proliferation in vivo. This regulation is of clinical significance, because progesterone administration promotes adult CM proliferation and improves cardiac function in MI in adult mice. Collectively, our results demonstrate that progesterone withdrawal may be a vital inducer of postnatal CM proliferative arrest; progesterone promotes both neonatal and adult cardiomyocyte proliferation by up‐regulating YAP expression.

## 2 MATERIALS AND METHODS

### 2.1 Animals experiments

All experimental procedures were approved by the Animal Care and Use Committee of the Third Military Medical University, and all animal experiments were performed to conform the National Institutes of Health (NIH) Guide for the Care and Use of Laboratory Animals. C57BL/6 mice and Sprague Dawley (SD) rats were supplied by the Experimental Animal Center of Daping Hospital, affiliated with the Third Military Medical University (Chongqing, China). In the neonatal mice studies, progesterone was intraperitoneally injected daily from postnatal day 1 (P1) to postnatal day 6 (P6); the hearts were harvested at P7 to analyse CM proliferation. Eight‐ to 10‐week‐old male C57Bl/6J mice were anaesthetized by inhalation of isoflurane (2%) and subjected to MI by ligation of the left anterior descending (LAD) coronary artery, as previously published.^12^ After the surgery, the mice were allowed to recover with free access to food and water for 48 hours and progesterone was intraperitoneally injected daily from day 2 until the heart was collected at day 7 and day 35 for analysis. The dose of progesterone for both neonatal and adult mice was 8 mg/kg/d, which has been used to investigate its neuroprotective effect in young adult and aged mice.^13^ Animals were euthanized using overdose of isoflurane (5%) and cervical dislocation before heart extraction.

### 2.2 Serum progesterone determination

Animals were euthanized using overdose of isoflurane (5%) before blood collection. Blood samples from embryonic day 16 (E16), P1, P7 and P14 mice were collected and placed at room temperature for 2 hours and then centrifuged at 300 *g* for 30 minutes to obtain the serum. The serum progesterone concentration was quantified using commercial progesterone ELISA kits (ADI‐900‐011, Enzo Life Sciences), following the manufacturer’s instructions. Briefly, 100 μL progesterone stands (5, 2.5, 1.25, 0.62, 0.31, 0.15 and 0.075 ng/mL) and diluted serum samples (1:10) were pipetted into 96‐well plates (anti‐mouse IgG‐coated) in duplicate. Then, 50 μL progesterone alkaline phosphatase conjugate and 50 μL solution of a monoclonal antibody to progesterone were added, gently mixed and incubated at room temperature on a plate shaker for 2 hours. After that, contents of the wells were removed and washed twice and 200 μL of the pNpp substrate solution was added to every well and incubated for 45 minutes at room temperature. Lastly, 50 μL of stop solution was added to each well, optical density was read immediately at 405 nm and progesterone concentration was calculated.

### 2.3 Echocardiographic evaluation

Similar to our previous study,^12^ 28 days after MI, mice were anaesthetized with isoflurane and cardiac function was determined by echocardiography, using GE Vivid 9‐Dimension Ultrasound. Two‐dimensionally guided M‐mode images of the short axis between the two papillary muscles were recorded to measure left ventricular (LV) wall structure and evaluate cardiac function by calculating LV fraction shortening (FS %) and LV ejection fraction (EF %). All measurements were carried out by a technician who was unaware of the experimental groups. The average of at least three measurements was used in all calculations.

### 2.4 Isolation and culture of neonatal rat cardiomyocytes

Neonatal CMs were isolated by enzymatic disassociation of hearts from 7‐day‐old SD rats according to our published procedure.^14^ Briefly, rats were euthanized using overdose of isoflurane (5%) and hearts were extracted. Then, hearts were washed with ice‐cold saline and minced into pieces smaller than 1 mm^3^ before enzymatic digestion using 1.25 mg/mL trypsin for 3 minutes. The heart pieces were collected further digested using 0.8 mg/mL collagenase II for 30 minutes at 37°C. The digestion supernatant was collected and added with an equal volume of Dulbecco’s modified Eagle’s medium (DMEM) supplemented with 10% FBS before being filtered through a cellular strainer. Then, cell suspension was incubated at 37°C for 90 minutes for differential attachment. After that, cells in the suspension were plated and cultured in DMEM with 10% foetal bovine serum at 37°C in atmosphere with 5% CO_2_ for 24 hours. Then, progesterone at various concentrations (10^−9^‐10^−4^ M) with or without the progesterone blocker RU486 (10^−6^ M) was added to the medium to incubate for 24 hours, and then, the cells were fixed with 4% polyformaldehyde for 20 minutes followed by immunostaining. The cells were collected and stored at −80°C for subsequent analysis of protein or mRNA expression. For small interfering RNA (siRNA) experiments, siRNAs against YAP mRNA were transfected into neonatal CMs using Lipofectamine 2000 transfection reagent (Invitrogen) for 24 hours prior to progesterone treatment. Sequences of two different siRNAs are as following: 1# si YAP, 5′‐GCCAUGAACCAGAGGAUCATT ‐3′; 2# si YAP, 5′‐GAUUGAAACAGCAGGAGUUTT‐3′.

### 2.5 Isolation of rod‐shaped cardiomyocytes

Rod‐shaped CMs were isolated from P7 and adult mice according to the protocol described previously^15^ and then being immunostained to measure cell size and evaluate CM proliferation. Briefly, mice were anaesthetized by intraperitoneal injection of pentobarbital sodium (120 mg/kg), and then, hearts were attached to the Langendorff apparatus and digested via retrograde perfusion of Tyrode solution containing collagenase B (1 mg/mL), trypsin (0.12 mg/mL) and CaCl_2_ (20 μmol/L). After about 10‐minute digestion, we minced the hearts into small pieces and pipetted the solution with a plastic Pasteur tube. Digestion was ceased with bovine serum albumin (BSA) (5 mg/mL), and then, cells equilibrated in Tyrode solutions were centrifuged down and fixed with 4% polyformaldehyde.

### 2.6 Immunostaining

The hearts were harvested and fixed with 4% polyformaldehyde, followed by dehydration and embedding into paraffin to cut 4‐μm‐thick sections. Fixed cells or tissue sections were permeabilized with Triton (0.1%) for 10 minutes and blocked in PBS with 5% BSA for 1 hours. Then, the samples were incubated with primary antibodies overnight at 4°C. After three washes with PBS, the samples were incubated with fluorescent secondary antibodies for 1 hour at room temperature, followed by 10 minutes of DAPI staining for nuclei visualization. After staining, the slides were mounted with Immu‐Mount and viewed under an Olympus confocal microscope (FluoView 1000). Cardiac troponin T (cTnT) and pan‐cadherin were immunostained to show intact CMs, and Ki67^+^, PH3^+^ and Aurora B^+^ CMs were counted to evaluate CM proliferation, as specified in the figure legends. All manual counts were performed by personnel who were unaware of the groupings and cell treatments. Primary antibodies used were as following: anti‐cardiac troponin T (MA5‐12960, Thermo Fisher Scientific), anti‐Ki67 (MA5‐14520, Thermo Fisher Scientific), anti‐PH3 (PA5‐17869, Thermo Fisher Scientific), anti‐Aurora B (ab2254, Abcam) and anti‐Pan‐cadherin (ab6529, Abcam). Secondary antibodies used were as following: Goat anti‐mouse IgG (H + L), Alexa Fluor Plus 488 (A‐32723, Thermo Fisher Scientific); Goat anti‐rabbit IgG (H + L), Alexa Fluor Plus 488 (A‐11035, Thermo Fisher Scientific).

### 2.7 WGA and Masson's trichrome staining

Tissue sections of P7 hearts were subjected to anti‐wheat germ agglutinin (WGA) immunofluorescence staining following the manufacturer’s protocol, and cross‐sectional area of the CMs was analysed as previously described.^14^ Briefly, after deparaffinization and rehydration, WGA was added to tissue sections and incubated for 30 minutes at room temperature. Then, images were taken with Olympus confocal microscope (FluoView 1000). Similar to previous studies,^16^ 80‐100 cells were randomly selected per section and four sections were measured per heart. Tissue sections from apex to base of post‐MI hearts were subjected to Masson’s trichrome staining following the manufacturer’s protocol to analyse the size of the fibrotic scars. Briefly, tissue sections were subjected to deparaffinization and rehydration, and incubation with Bouin’s solution at room temperature overnight. Then, the slides were washed with distilled water and sequentially stained with working haematoxylin for 15 minutes, Biebrich scarlet for 5 minutes, phosphotungstic/phosphomolybdic acid for 10 minutes, aniline for blue for 5 minutes and 1% acetic acid solution for 3 minutes. Lastly, images were acquired to quantify scar size using ImageJ software (Wayne Rasband, NIH), which was calculated as relative to total left ventricle wall size.

### 2.8 Real‐time RT‐PCR

Total RNAs of cells or tissues were extracted using RNAiso Plus (9109, TAKARA), following standard protocols. The extracted RNA was reverse‐transcribed (RT‐PCR) with the TOYOBO RT‐PCR Kit. Quantitative real‐time PCRs of GAPDH, cell cycle genes CCNA1, CDK1, CDK4, CCNB1, CCND1, YAP and YAP target genes ANKRD1, CYR61 and CTGF, and regulators of CM proliferation NRG1, Postn, GATA4, TBX20 and FSTL1 were carried out. Bio‐Rad software was utilized to analyse melting curves, and a comparative cycle threshold method was used to calculate relative mRNA expression. All experiments were repeated at least three times, and the expression of mRNA was normalized by GAPDH. The primers used are listed in Table 1.

**Table 1:** PCR primers used for RT‐PCR

Primer name	Species	Sequence
CCNA1 forward	Mouse	AAGAACCTGAGAAGCAGGGC
CCNA1 reverse	Mouse	CTAGCACGGTTCTCTGTGGG
CCNA1 forward	Rat	TCGTGTCTGTTGGGGGAACC
CCNA1 reverse	Rat	CGACTCCACTCTTCGAGCTGT
CDK1 forward	Mouse	ACGGCTTGGATTTGCTCTCA
CDK1 reverse	Mouse	ACGATCTTCCCCTACGACCA
CDK1 forward	Rat	GGAACAGAGAGGGTCCGTTG
CDK1 reverse	Rat	AGAGATTTCCCGGATTGCCG
CCNB1 forward	Mouse	TCTCGAATCGGGGAACCTCT
CCNB1 reverse	Mouse	GCCATACTGACCTTGGCCTT
CCNB1 forward	Rat	GGGTGTCTTCTCAGATCGGC
CCNB1 reverse	Rat	GTTCTCGATCTCAGCAGGGG
CDK4 forward	Mouse	ACTCGATATGAACCCGTGGC
CDK4 reverse	Mouse	AGCACAGACATCCATCAGCC
CDK4 forward	Rat	TGAGGGGGCCTCTCTAGCTC
CDK4 reverse	Rat	CCATAGGCACCGACACCAAT
Ankrd1 forward	Rat	AGACTAACGGCTGCCAACAT
Ankrd1 reverse	Rat	TTCCGTTGTTCTTCCCCCAG
CYR61 forward	Rat	TTGTTGGTTCTGTGTCGCCG
CYR61 reverse	Rat	CCTGGTCAAGTGGAGAAGGG
CTGF forward	Rat	GTCTCGCCGCCCTTCTTATTA
CTGF reverse	Rat	ACTGGGAGTTTCATTCGCCA
YAP forward	Rat	TTTCGGCAGGCAATACGGAA
YAP reverse	Rat	AGCTAATTCCCGCTCTGACG
NRG1 forward	Rat	GGTGGTGATCGAGGGAAAGG
NRG1 reverse	Rat	CACCTTGACCAGATAGGGCG
Postn forward	Rat	TGCAAAAAGACACACCTGCAA
Postn reverse	Rat	CCGAAGTCAATGGGGCTCTT
GATA4 forward	Rat	CAACACTCCCCTTGAGGCAT
GATA4 reverse	Rat	GAAGGGAAGGCACCTCTGAC
TBX20 forward	Rat	GCATAAGTACCAGCCGAGGG
TBX20 reverse	Rat	ACCTACACTTTGCAGAACAAGA
FSTL1 forward	Rat	CGATGAGAACGCTGACTGGA
FSTL1 reverse	Rat	AGGAGGGTTGAAGGATGGGT

### 2.9 RNA sequencing

We isolated and cultured P7 CMs using 7‐day‐old SD rats and treated cells with/without progesterone (10^−7^ M) for 24 hours to prepare sample (three biological replicates for each group) for RNA sequencing (RNA‐Seq). Libraries were constructed with the NEBNext Ultra™ RNA Library Prep Kit for the Illumina system according to the manufacturer’s instructions, and sequencing was performed using the Illumina HiSeq platform by the Novogene Bioinformatics Institute (Beijing, China). GO and KEGG pathway analysis were performed using the OmicShare tools, a free online platform for data analysis (http://www.omicshare.com/tools). The hierarchical clustering heatmap was generated with the ggplot library.

### 2.10 Immunoblotting

Protein was extracted using RIPA buffer (0.1% SDS, 1% Nonidet P‐40, 150 mmol/L NaCl, 0.5% sodium deoxycholate, 50 mmol/L Tris and protease inhibitor mixture; Roche). Protein concentrations were measured with the enhanced BCA Protein Assay Kit (Proteintech Group Inc). Samples (50 μg) were subjected to SDS‐PAGE with 10% polyacrylamide gel followed by electrotransfer into polyvinylidenedifluoride membranes and then blocked with Tris‐buffered saline (TBS), containing 5% non‐fat dry milk for 1 hour. The membranes were incubated with primary antibodies overnight at 4°C, washed with TBS three times and then incubated with the secondary antibody at room temperature for 1 hour. The Odyssey Infrared Imaging System (Li‐COR Biosciences) was used to visualize the bands, and Quantity One image analysis software was used to analyse the relative intensities of the protein bands. GAPDH was used to normalize the densitometric intensity for proteins. Antibodies used were as following: anti‐Ki67 (MA5‐14520, Thermo Fisher Scientific), anti‐PH3 (PA5‐17869, Thermo Fisher Scientific), anti‐Aurora B (ab2254, Abcam), anti‐YAP (ab205270, Abcam) and anti‐GAPDH (ab8245, Abcam).

### 2.11 Luciferase assay

Primary neonatal CMs were cultured in 24‐well plates and transfected with luciferase reporter plasmids, including pGL3‐basic (firefly luciferase reporter), pRL‐TK (*Renilla* luciferase reporter) and YAP‐p (firefly luciferase reporter with YAP promoter), using Lipofectamine 2000 transfection reagent (Invitrogen), following the manufacturer’s instructions. After transfected for 24 hours, the medium was removed and the cells were incubated in serum‐free medium and treated with or without progesterone (10^−7^ M) for another 24 hours. Then, cells were lysed using lysis buffer (50 mmol/L Tris‐HCl (pH 7.8), 50 mmol/L Na‐2‐(N‐Morpholino) ethanesulfonic acid (MES) (pH 7.8), 0.2% Triton X‐100 and 1 mmol/L dithiothreitol) at room temperature for 10 minutes and centrifuged at 10 000 *g* for 5 minutes. 100 µL of firefly luciferase assay buffer and *Renilla* luciferase assay buffer was added to 100 µL of supernatant and gently mixed prior to measurement of firefly luminescence and *Renilla* luminescence in BK‐L96C luminometer (Biosino). Firefly luciferase/*Renilla* luciferase ratio was used to measure promoter activity.

### 2.12 ChIP‐qPCR

Chromatin immunoprecipitation (ChIP), coupled with quantitative PCR (qPCR), was performed to examine the interaction of the progesterone receptor with the YAP promoter, using a Chromatin Immunoprecipitation Assay Kit (17‐371, Millipore), according to the manufacturer’s instructions. Briefly, neonatal CMs were treated with progesterone (10^−7^ M) for 24 hours and cross‐linked with formaldehyde (1% in PBS) for 10 minutes, and the reaction was quenched with glycine (0. 125 M) for 5 minutes. Then, the cells were lysed in SDS buffer and chromatin was fragmented to 180 bp‐360 bp using EZ‐Zyme™ Kit (17‐295, Millipore) according to the manufacturer’s instructions. Chromatin was precipitated with progesterone receptor antibody (ab2765, Abcam), and the immunoprecipitates (or no IP input) were treated with RNase A and proteinase K for purification of DNA. The purified DNA fragments were analysed by qPCR using three pairs of primers for YAP gene promoter (within 2000 bp upstream of YAP transcription start site). The results were expressed as enrichment relative to input.^17^ The primers used are listed in Table 2.

**Table 2:** PCR Primers used for ChIP‐PCR

YAP‐1#	Forward	TGTTAAAACGCGGACCGGAT
YAP‐1#	Reverse	CCAGAAATCCCGACAGCCC
YAP‐2#	Forward	CGAAGATTGTCCGGAGTTGGA
YAP‐2#	Reverse	CAAAACACTTGAGGGCGGGT
YAP‐3#	Forward	TCACACTGCAGACAAAATCATTAAC
YAP‐3#	Reverse	TCCCTTGTTGAAGTCATGAGGTA

### 2.13 Electrophoretic mobility shift assay

Electrophoretic mobility shift assay (EMSA) was performed as previously published.^18^ Nuclear extracts were prepared using NE‐PER™ Nuclear and Cytoplasmic Extraction Kit (78835, Thermo Fish Scientific), following standard procedures. Briefly, cells were harvested using trypsin‐EDTA and centrifuged at 500 *g* for 5 minutes. Ice‐cold CER I was added to cell pellet and vortexed vigorously to fully suspend prior to addition of CER II. Then, the tube was vortexed for 5 seconds vigorously and centrifuged for 5 minutes at 16 000 *g*. Lastly, supernatant (cytoplasmic extract) was removed and ice‐cold NER was added to lyse nuclear pellet to obtain nuclear protein. DNA probes representing fragments of the YAP promoter were synthesized and 5′ end‐labelled with DyLight 800. In addition, control non‐labelled DNA probes were used as competitors. The extracted proteins were pre‐incubated with progesterone receptor antibody (ab2765, Abcam) or IgG before being incubated with labelled probe (50 nmol/L) or control non‐labelled DNA probes at 200‐fold molar excess. Then, the protein‐bound and free DNAs were separated on 6% polyacrylamide gel in the dark. The bands in the gels were visualized using Odyssey Infrared Imaging System (Li‐COR Biosciences). The sequence of the DNA probe was 5′‐AACTATTTTTTGTTCTGTACCTCATGACTT‐3′.

### 2.14 Statistical analysis

All data are expressed as means ± SEM, and statistical analysis was performed using SPSS 18.0 statistical package (International Business Machines Corporation, New York, USA). Significant differences between and among groups were determined by one‐way ANOVA and Student‐Newman‐Keuls post hoc test for groups >2 or Student’s *t* test for groups = 2. *P* < .05 was considered significant.

## 3 RESULTS

### 3.1 Progesterone promotes postnatal CM proliferation in vivo

**Figure 1:**
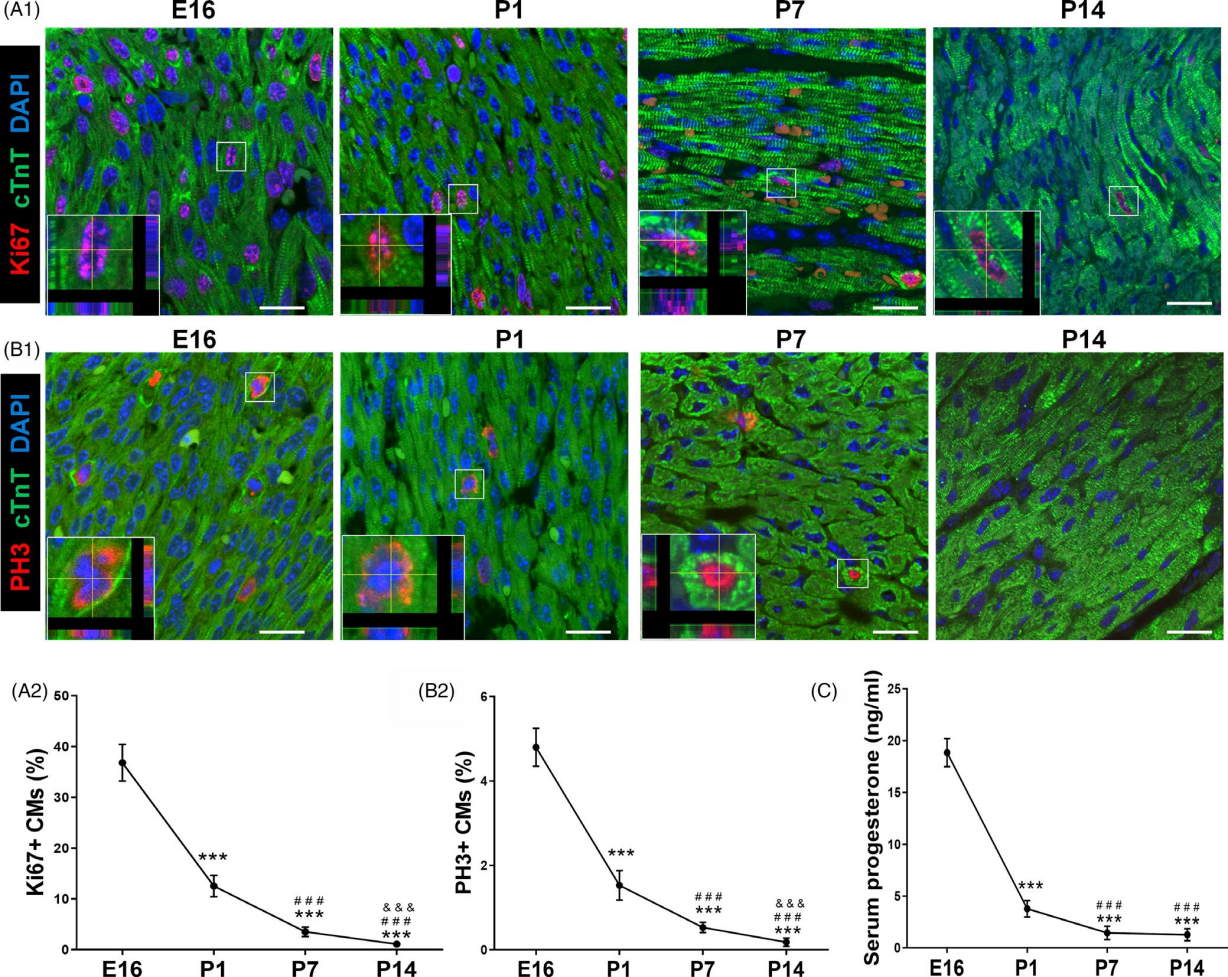
Serum progesterone concentration declines after birth and is associated with decrease in CM proliferation. A,B, Evaluation of CM proliferation by Ki67 and PH3 immunostaining at E16, P1, P7 and P14 hearts. Representative images with z‐stacking are shown in A1 and B1, and quantification of percentages of Ki67^+^ and PH3^+^ CMs is shown in A2 and B2, respectively (n = 5). Scale bars are 20 µm. cTnT indicates cardiac troponin T. C, Serum concentrations of progesterone in mice at E16, P1, P7 and P14 detected using *ELISA* kit (n = 5‐8). ^***^ *P* < .001 vs E16; ^###^ *P* < .001 vs P1; ^&&&^ *P* < .001 vs P7. CM, cardiomyocyte

To investigate the relationship between serum progesterone and CM proliferation, serums and hearts were harvested in E16, P1, P7 and P14 mice. As shown in Figure 1A,B, CM (labelled by cTnT, a specific marker of cardiomyocyte) proliferation, determined by cell cycling marker Ki67 and G2‐M progression marker PH3 staining, was decreased, along with the age‐related decrease in serum progesterone level (Figure 1C).

**Figure 2:**
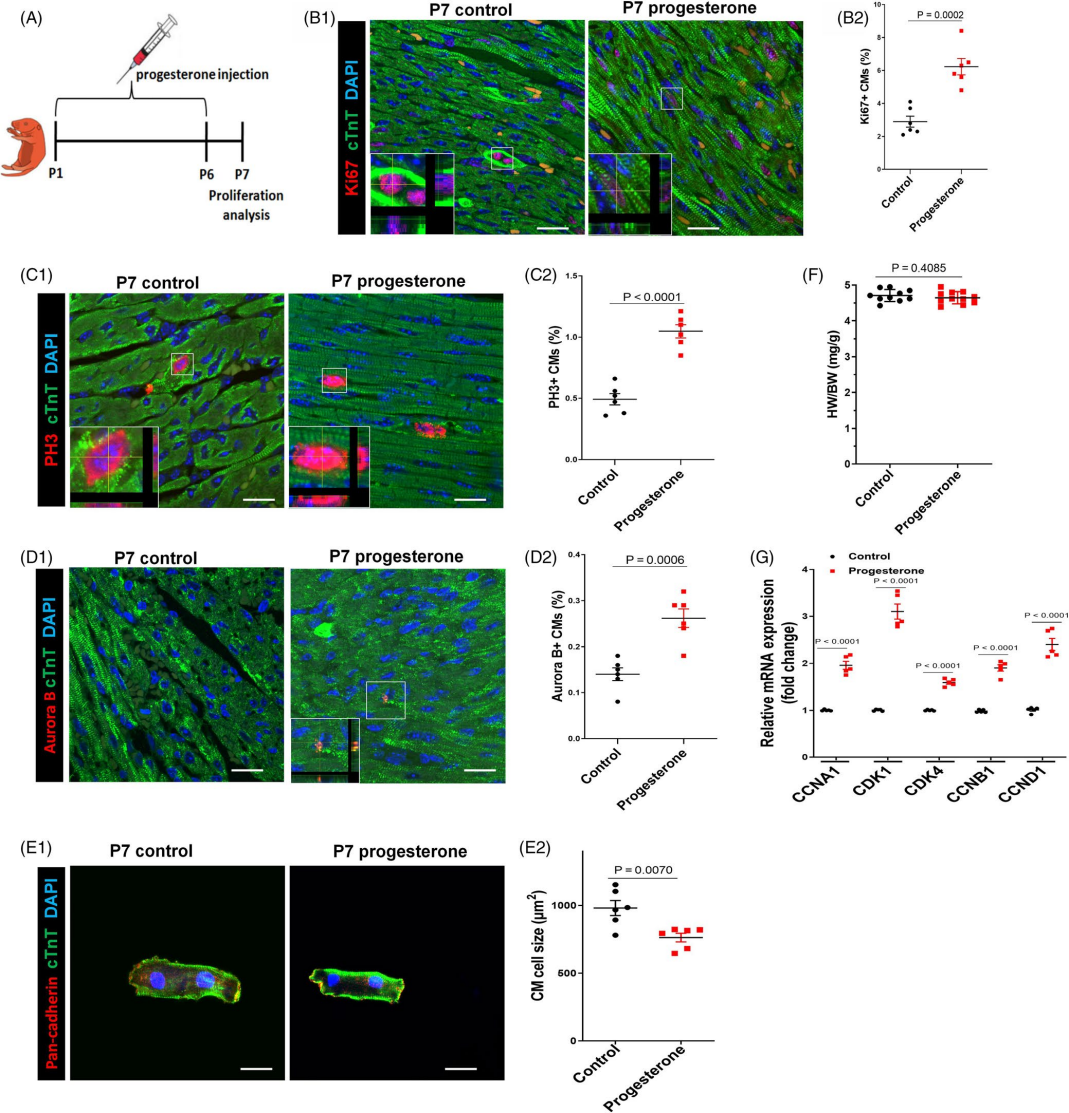
Progesterone supplementation promotes postnatal CM proliferation in vivo. A, Overview of the experimental set‐up of the daily intraperitoneal injection of progesterone (8 mg/kg) or control vehicle (corn oil) from P1 to P6. Hearts were harvested at P7. B‐D, CM proliferation was evaluated by Ki67, PH3 and Aurora B immunostaining. Representative images with z‐stacking are shown in B1, C1 and D1, and quantification of percentages of Ki67^+^, PH3^+^ or Aurora B^+^ CMs is shown in B2, C2 and D2, respectively (n = 6). Scale bars are 20 µm. E, Representative images (E1) and quantification (E2) of the cell size of CMs (labelled by cTnT and pan‐cadherin to show intact cardiomyocytes) isolated from P7 heart. Eighty to one hundred cells were randomly selected per heart (n = 6). Scale bars are 20 µm. F, Heart weight and body weight (HW/BW) ratio (n = 10‐11). G, The mRNA expression of cell cycle activators in mouse hearts was analysed by real‐time PCR (n = 5). CM, cardiomyocyte

To test, directly, the role of progesterone in the decline in CM proliferation after birth, neonatal mice were injected daily with progesterone or vehicle during postnatal periods, P1 to P6 (Figure 2A). Remarkably, postnatal progesterone supplementation increased the P7 CM proliferation, evidenced by the increase in immunostaining of Ki67^+^, PH3^+^ and cleavage furrow–localized cytokinesis marker Aurora B^+^ CMs (Figure 2B‐D). Progesterone's cardiomyocyte proliferative effect was not different between genders (Figures S1 and S2). We found progesterone‐treated CM sizes were significantly smaller than control ones (Figure 2E1 E2, Figure S3A1,A2), though there was no significant difference for heart sizes between progesterone and control groups (Figure 2F), indicating that progesterone‐treated hearts had more CMs. Because the decrease in the expression of cell cycle genes in postnatal period plays a vital role in cell cycle exit,^19^ we also measured the effect of progesterone on the expression of cell cycle genes by qPCR. Notably, cell cycle genes, including CCNA1, CDK1, CDK4, CCNB1 and CCND1, were up‐regulated by progesterone treatment (Figure 2G). These data suggest that progesterone supplementation could promote postnatal CM proliferation in vivo.

### 3.2 Progesterone promotes CM proliferation in vitro via a progesterone receptor‐dependent manner

**Figure 3:**
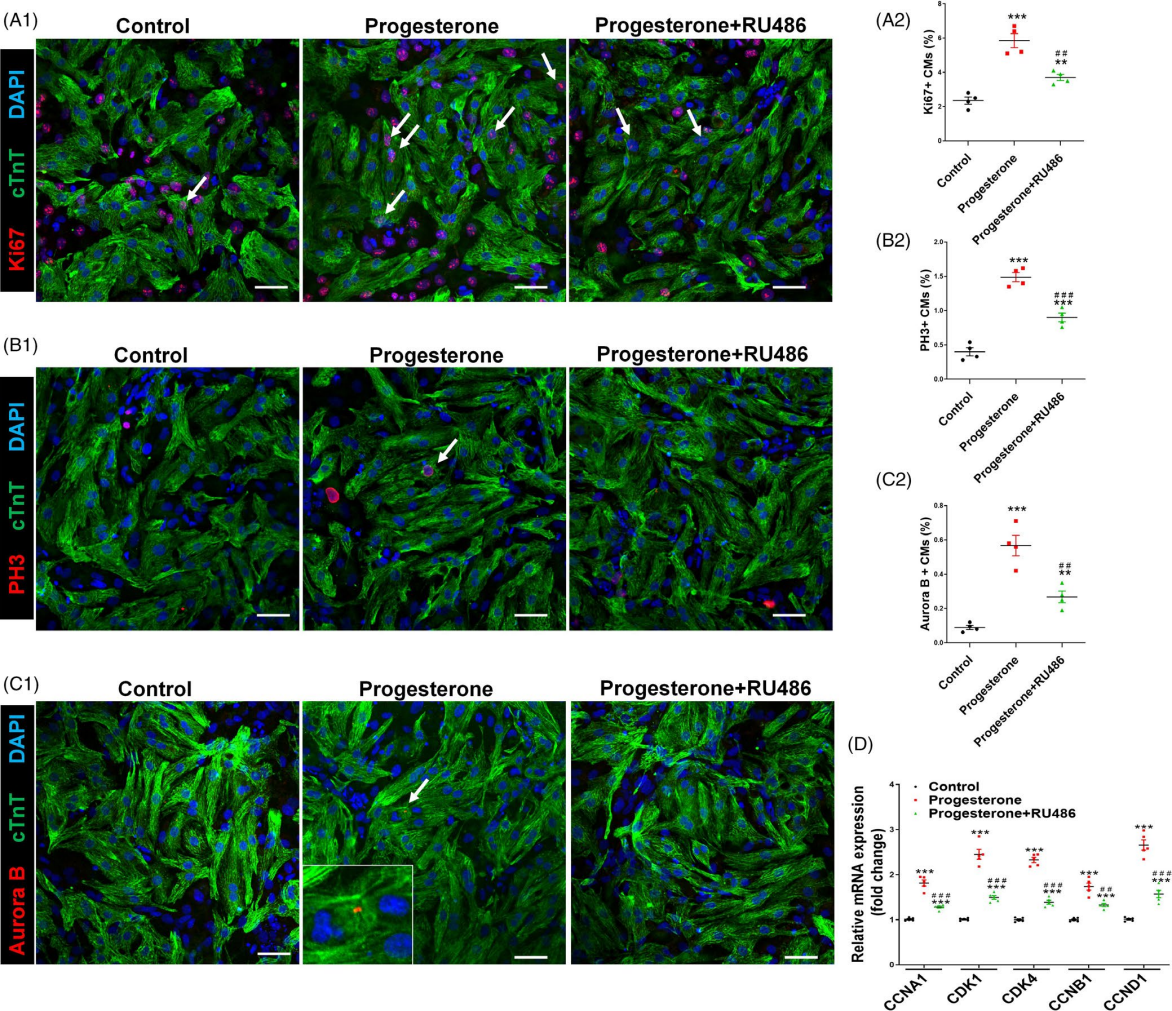
Progesterone promotes CM proliferation in vitro in a progesterone receptor‐dependent manner. Cultured P7 CMs were treated with control vehicle (DMSO) or progesterone (10^−7^ M), alone or in combination with the progesterone receptor inhibitor RU486 (10^−6^ M). CM proliferation was analysed 24 h after progesterone stimulation. A‐C, CM proliferation was evaluated by Ki67, PH3 and Aurora B immunostaining. Representative images are shown in A1, B1 and C1, and quantification of percentages of Ki67^+^, PH3^+^ or Aurora B^+^ CMs is shown in A2, B2 and C2, respectively. (3000‐5000 cells were randomly selected and analysed for each group, and four independent experiments were conducted). Scale bars are 40 µm. D, The mRNA expression of cell cycle activators in P7 CMs was analysed by quantitative PCR (n = 5). (^**^ *P* < .01, ^***^ *P* < .001) vs Control; (^##^ *P* < .01, ^###^ *P* < .001) vs Progesterone. CM, cardiomyocyte

**Figure 4:**
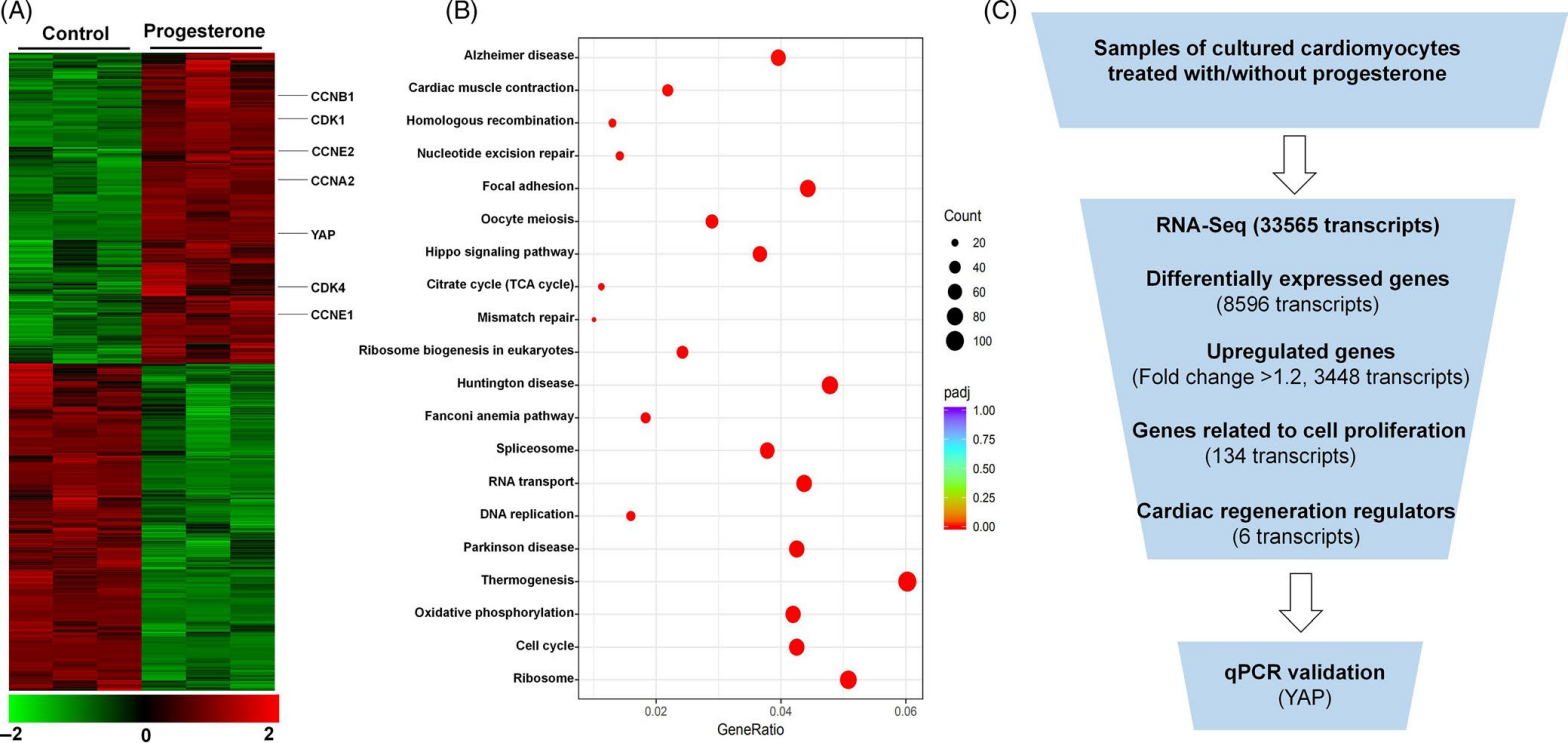
RNA‐Seq profiles of P7 CMs treated with or without progesterone. A, Heatmap of genes whose expression was significantly different between control and progesterone‐treated CMs. Selected genes including YAP and cell cycle genes are indicated. The colour chart indicates fold change of expression using a log2 scale. B, KEGG pathway analysis of up‐regulated genes. Significantly enriched pathways of up‐regulated genes are shown. C, Selection strategy of cardiac regeneration regulators involved in progesterone‐induced CM proliferation from RNA‐Seq profiling. CM, cardiomyocyte; YAP, yes‐associated protein

To uncover the mechanism underlying the progesterone‐induced CM proliferation, we studied the effect of progesterone in in vitro experiments. Neonatal CMs were isolated from 7‐day‐old rats and treated with progesterone at various concentrations (10^−9^‐10^−4^ M) for 24 hours. The results showed that progesterone promoted CM proliferation in a concentration‐dependent manner, as detected by Ki67 and PH3 staining, peaking at 10^−8^ and 10^−7^ M; the proliferative effect started to decline at 10^−6^ M and returned to control values at 10^−4^ M (Figure S4A,B). Therefore, 10^−7^ M progesterone was used in the in vitro experiments. Being a steroid hormone, progesterone regulates target cell function by binding to its receptor, resulting in the promotion of progesterone‐responsive gene expression.^20^ Thus, we tested whether progesterone could promote CM proliferation via a receptor‐dependent manner. As is shown in Figure 3A‐C and S5, progesterone significantly increased the percentage of Ki67^+^, PH3^+^ and Aurora B^+^ CMs, and also total protein levels of these proliferation markers; this effect was blocked by co‐treatment with the progesterone receptor inhibitor RU486. Furthermore, RU486 co‐treatment also prevented the progesterone‐induced up‐regulation of the expression of cell cycle genes (Figure 3D). Taken together, these results support the notion that progesterone promotes CM proliferation in a progesterone receptor‐dependent manner. In addition, we also performed RNA‐Seq on cultured P7 CMs with or without progesterone treatment to further confirm proliferative effect of progesterone and uncover underlying mechanism, and the results further confirmed up‐regulation of cell cycles genes, including CDK1, CDK4 and CCNB1 (Figure 4A). KEGG pathway analysis of up‐regulated genes indicated increased cell cycle activities in progesterone group (Figure 4B).

### 3.3 Progesterone promotes CM proliferation through up‐regulation of YAP expression

**Figure 5:**
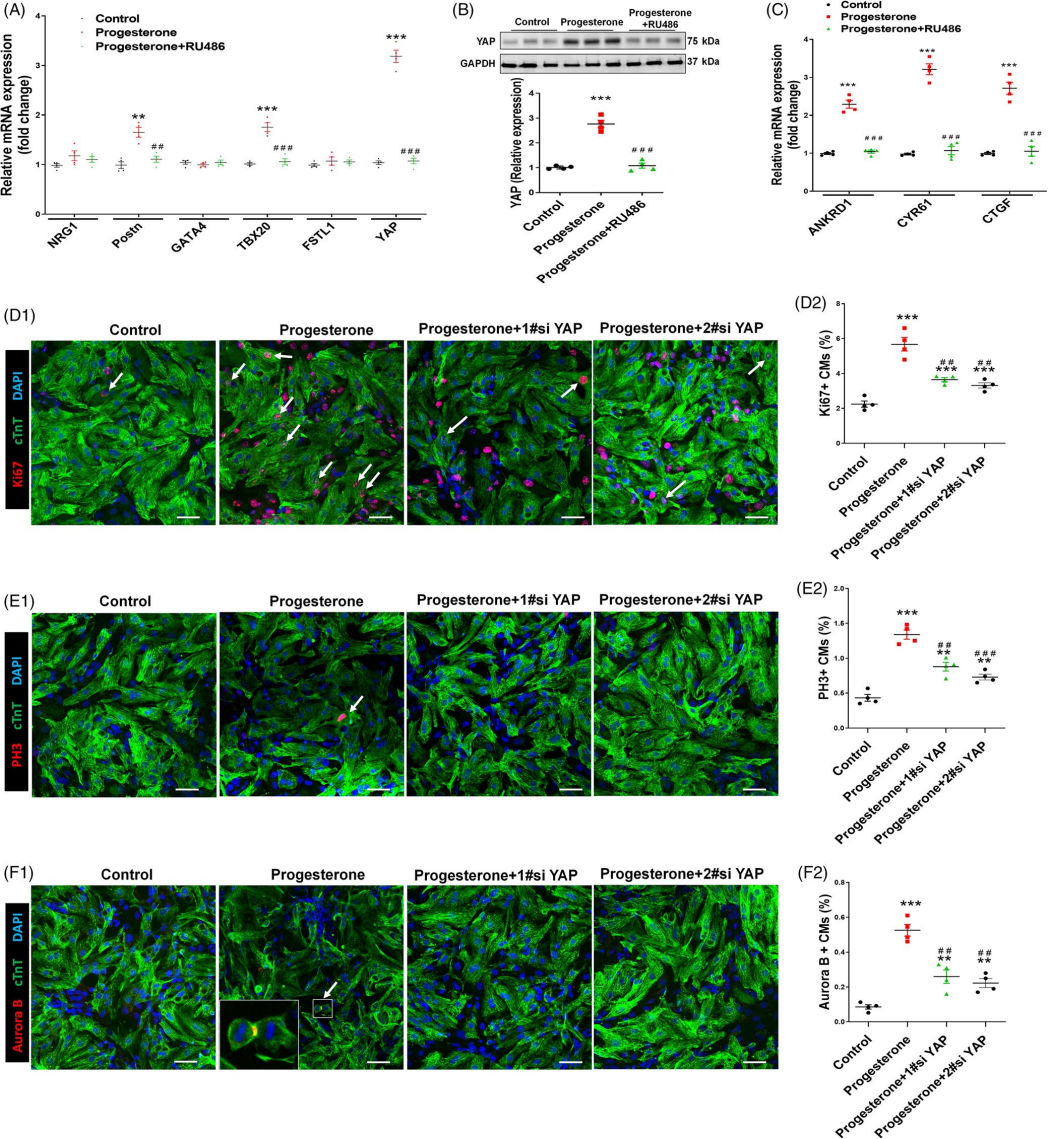
Progesterone promotes CM proliferation through up‐regulation of YAP expression. Cultured P7 CMs were treated with control (vehicle, DMSO) or progesterone (10^−7^ M), alone or in combination with RU486 (A‐C) or YAP siRNAs (D‐F). A, Proliferation regulator mRNA expressions of CM were analysed by quantitative PCR (n = 4). B, Representative immunoblots (B1) and quantification (B2) of YAP protein expression were analysed by Western blotting (GAPDH was used as internal control) (n = 4). C, The mRNA expression of YAP target genes ANKRD1, CYR61 and CTGF was analysed by quantitative real‐time PCR (n = 4). D‐F, CM proliferation was evaluated by Ki67, PH3 and Aurora B immunostaining. Representative images are shown in D1, E1 and F1, and quantification of percentages of Ki67^+^, PH3^+^ or Aurora B^+^ CMs is shown in D2, E2 and F2, respectively (3000‐5000 cells were randomly selected and analysed for each group) (n = 4). Scale bars are 40 µm (^**^ *P* < .01, ^***^ *P* < .001) vs Control; (^##^ *P* < .01, ^###^ *P* < .001) vs Progesterone. CM, cardiomyocyte; YAP, yes‐associated protein

The RNA‐Seq showed that there were 8596 transcripts differentially expressed between control and progesterone‐treated CMs, with 3448 transcripts up‐regulated (fold change > 1.2). Cardiac regeneration regulators, involved in progesterone‐induced CM proliferation, were selected from RNA‐Seq profiling (Figure 4C). Among the up‐regulated genes, there were 134 cell cycle–related transcripts (Figure 4C). And six of the 134 transcripts, including NRG1, Postn, GATA4, TBX20, FSTL1 and YAP, were reported as key regulators for cardiac regeneration and cell cycle gene expression.^21^ Based on qPCR‐validated results, we found YAP was the one with the biggest changed fold after progesterone treatment (Figure 5A); after blockade of progesterone receptor by its inhibitor RU486, the stimulatory effect of progesterone on YAP expression was reduced (Figure 5A,B). The progesterone‐induced up‐regulation of YAP protein was associated with its functional activation, because progesterone also up‐regulated YAP target genes, including ANKRD1, CYR61 and CTGF (Figure 5C). These results indicated that up‐regulation of YAP expression may be the underlying mechanism through which progesterone promotes CM proliferation. YAP silencing study further confirmed the importance of YAP in the progesterone‐mediated CM proliferation, because down‐regulation of YAP expression by YAP‐specific siRNA (Figure S6) abolished the pro‐proliferative effect of progesterone, evidenced by the decrease in percentage of Ki67^+^, PH3^+^ and Aurora B^+^ CMs and also total protein levels of these proliferation markers (Figure 5D‐F and Figure S7). These findings suggest that progesterone promotes CM proliferation through up‐regulation of YAP expression.

**Figure 6:**
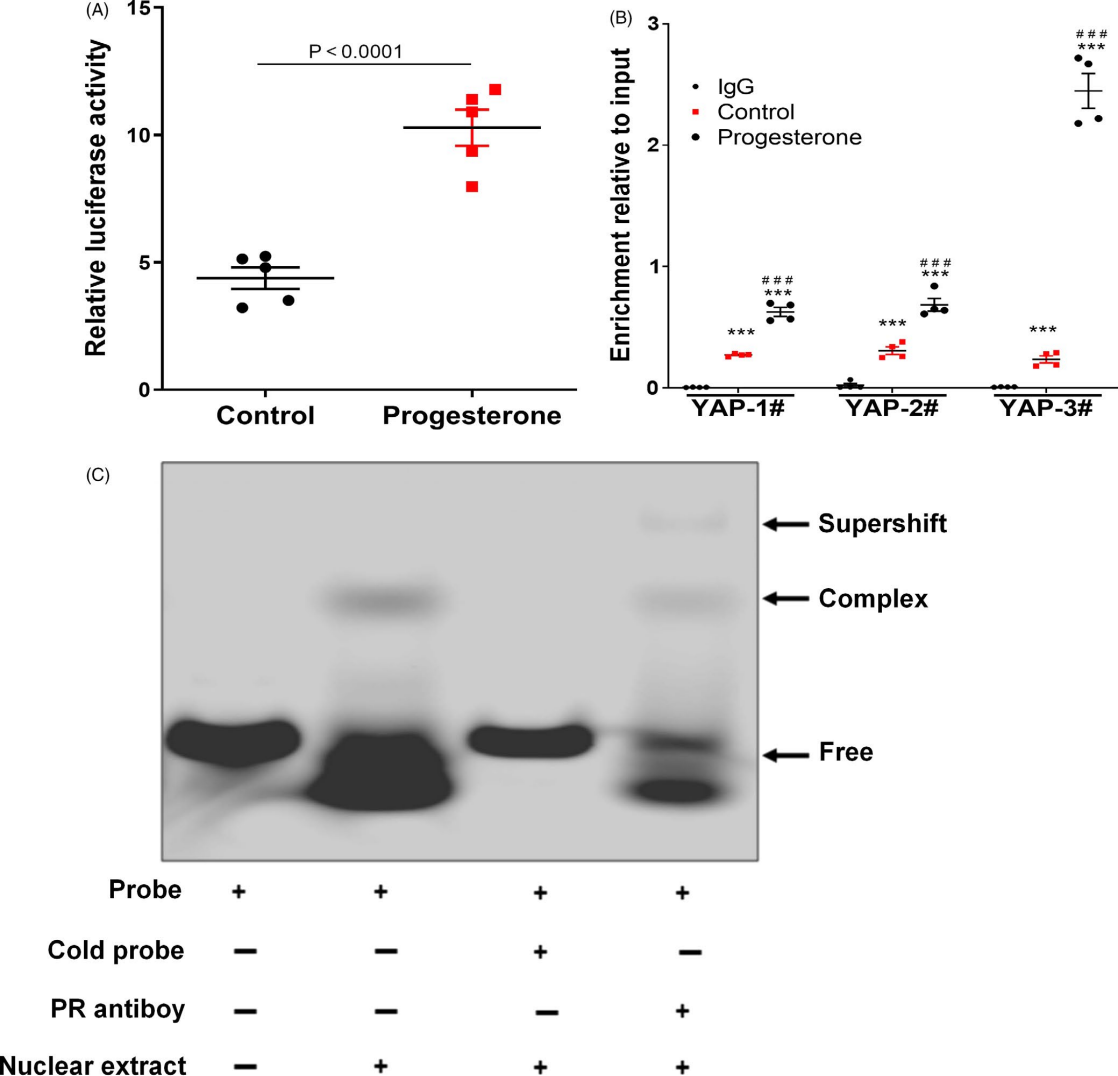
Progesterone promotes YAP expression by progesterone receptor‐mediated activation of YAP promoter in CM. A, YAP luciferase reporter activity assay. The firefly luciferase/*Renilla* luciferase ratio in cell lysates was calculated and expressed as relative luciferase activity (*P* < .0001 vs Control, n = 5). B, Binding of progesterone receptor on YAP promoter was detected by ChIP‐qPCR. DNA/protein complexes of cells treated with or without progesterone were captured by progesterone receptor antibody, and IgG was used as negative control. The immunoprecipitates were further analysed by quantitative PCR (qPCR) using three pairs of primers for YAP gene promoter (n = 4). ^***^ *P* < .001 vs IgG; ^###^ *P* < .001 vs Control. C, EMSA assay. Nuclear extracts of NRVMs were incubated with DyLight 800‐labelled DNA oligonucleotide probes containing YAP promoter fragments to show the binding of probe with the corresponding protein in the nuclear extracts; 200‐fold unlabelled probe as competitor was taken as negative control; progesterone receptor antibody was used to show the mobility super‐shift. CM, cardiomyocyte; YAP, yes‐associated protein

As progesterone receptor is a transcription factor to regulate target gene expression,^22^ we, therefore, investigated the effect of progesterone on YAP trans‐activation by performing luciferase reporter activity assay. The results showed that progesterone stimulation significantly increased luciferase activity of YAP promoter (Figure 6A). The progesterone receptor deposition on the YAP promoter was further determined by ChIP assays. We found that progesterone receptor was present at all three regions of the YAP promoter and progesterone increased the enrichment of the binding (Figure 6B). Sequence analysis identified a canonical progesterone receptor‐responsive element located at −1625 bp to −1620 bp of YAP promoter in rat (Figure S8). We also determined whether progesterone physically binds to the YAP promoter by performing EMSA in CMs using DyLight 800‐labelled oligonucleotide probe containing YAP promoter element in vitro. As shown in Figure 6C, probe and nuclear extract could effectively produce DNA‐protein complex band, which was made invisible by adding 200‐fold molar excess unlabelled probe, and the presence of progesterone receptor antibody super‐shifted the DNA‐protein complex, demonstrating that the progesterone receptor specifically binds to the YAP promoter. Collectively, these results suggest that progesterone increased YAP expression through the progesterone receptor by binding directly with the YAP promoter and consequently promoting YAP transcription in CMs.

### 3.4 Progesterone promotes adult CM proliferation and improves cardiac function in MI

**Figure 7:**
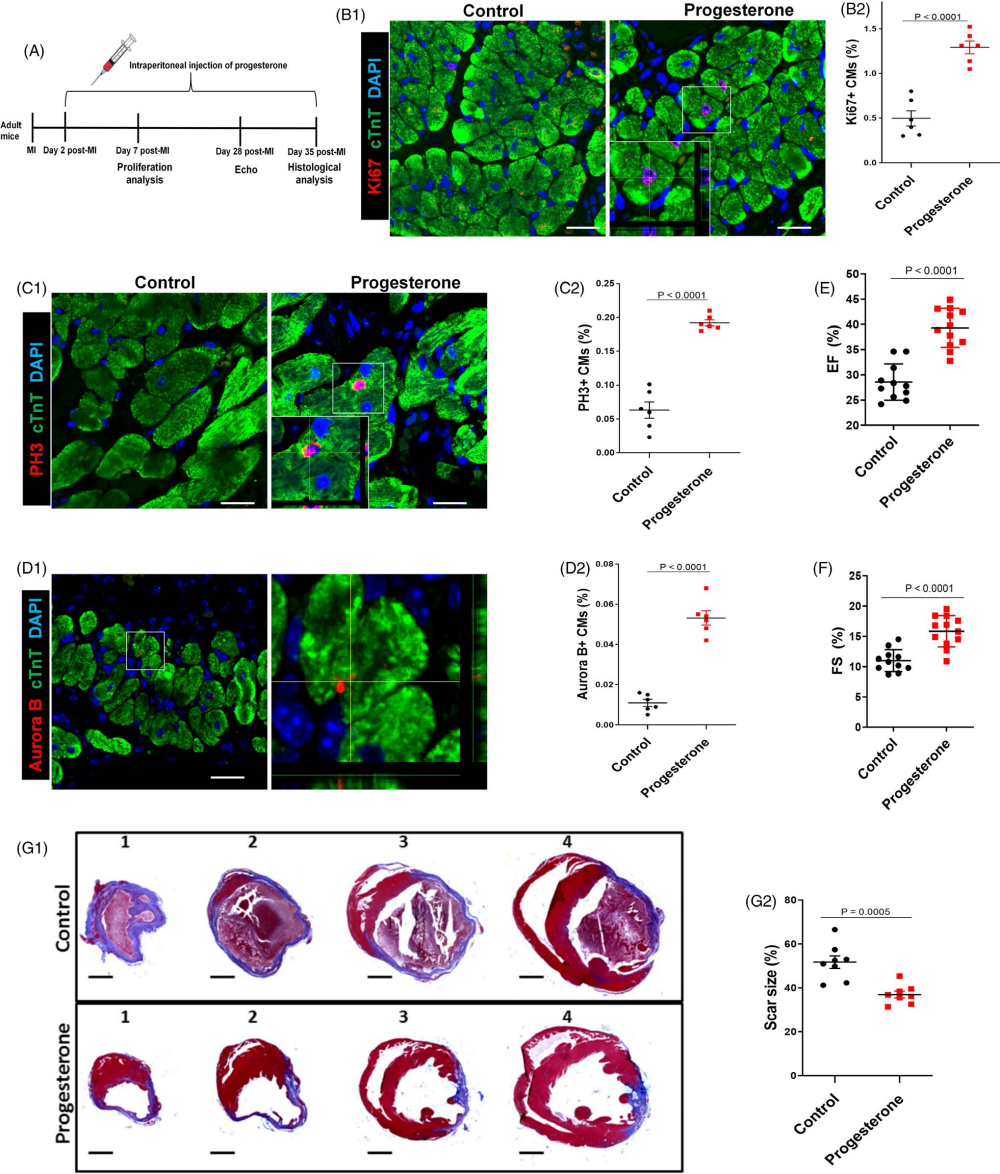
Progesterone promotes adult CM proliferation and improves cardiac function after MI. A, Adult mice were subjected to MI by ligation of the left anterior descending coronary artery and intraperitoneally injected daily with progesterone (8 mg/kg) or control vehicle (corn oil) from day 2 to day 35 post‐MI. B‐D, Hearts were harvested at day 7 post‐MI; CM proliferation in the peri‐infarcted area was evaluated by Ki67, PH3 and Aurora B staining in CMs. Representative images with z‐stacking and quantification of percentages of those markers are shown (more than 5000 cells were randomly selected in each heart, and 6 mice were analysed in each group). Scale bars are 20 µm. E,F, Echocardiographic (Echo) analyses of the effect of progesterone on cardiac function were performed 28 days after MI. Quantitative analysis of left ventricular ejection fraction (EF) and left ventricular fraction shortening (FS) is shown (n = 11‐12). G, Hearts were harvested at day 35 post‐MI. Representative images (G1) and quantification (G2) of fibrotic scar of heart sections were analysed using Masson’s trichrome staining (n = 8). Scale bars are 1 mm. CM, cardiomyocyte; MI, myocardial infarction

Finally, we assessed the effect of progesterone on adult CM proliferation and explored the clinical translational potential of the above studies. MI in adult mice was induced by ligation of the LAD coronary artery, followed by the daily intraperitoneal injection of progesterone (Figure 7A). At day 7 after MI, the percentages of Ki67^+^, PH3^+^ and Aurora B^+^ CMs in the peri‐infarcted area were significantly higher in the progesterone‐ than the vehicle‐treated group, indicating that progesterone could promote adult CM proliferation after MI (Figure 7B‐D). Immunostaining of isolated CMs further showed that percentages of Ki67^+^ and PH3^+^ CMs were significantly higher in the progesterone‐ than the vehicle‐treated group (Figure S9A,B). Consistent with in vitro results, progesterone also significantly increased YAP expression in CMs after MI (Figure S10). At day 28 after MI, cardiac function was measured by echocardiography. As shown in Figure 7E,F, progesterone was also able to ameliorate the loss of cardiac function induced by MI, evidenced by the increase in LVEF and LVFS. We also examined cardiac fibrosis at day 35 after MI by Masson’s trichrome staining and found that progesterone treatment significantly decreased the fibrotic scars (Figure 7G). These findings suggest that progesterone can promote adult CM proliferation and cardiac repair after MI.

## 4 DISCUSSION

In recent years, the concept that adult mammalian CM is completely incapable of regeneration has been challenged by many studies.^21^, ^23^ Indeed, increasing pieces of evidence have demonstrated that adult mammalian CM undergoes a very low level of proliferation, but this is insufficient to compensate for CM loss and restore cardiac function after injury.^24^, ^25^ Thus, understanding the mechanisms by which CMs exit the cell cycle shortly after birth and how to stimulate endogenous CM proliferation represent a promising therapeutic strategy for cardiac regeneration and repair. Here, we report that the postnatal decrease in circulating progesterone is an important factor for the postnatal cell cycle exit of the CM and progesterone supplementation prolongs the CM postnatal proliferative and regenerative window. Importantly, progesterone also promotes adult CM proliferation and improves cardiac function post‐MI.

The upstream signals that cause the postnatal cell cycle exit of the CM are largely unknown, for which considerable efforts have been recently devoted to understand this phenomenon. Recent studies have shown that the transition from hypoxic circulation in the foetus to the oxygen‐rich postnatal circulation induces CM cell cycle arrest through DNA damage response; postnatal hypoxaemia can prolong the postnatal CM proliferative window and enhances adult CM proliferation.^26^, ^27^ These studies imply that birth‐induced change of the circulatory environment from foetal to postnatal life may be key factors that account for the CM cell cycle exit. In support of this notion is a recent study showing that the loss of cardiac regenerative capacity in adult mammals is, to some extent, triggered by the postnatal increase in thyroid hormones.^28^ Our results show that serum progesterone and CM proliferation declined in parallel and postnatal progesterone supplementation significantly promoted neonatal CM proliferation both in vivo and in vitro. Thus, together with above studies, we propose that circulatory environmental shift, especially the hormonal environment, may represent the underlying mechanism for the postnatal loss of mammalian cardiac regenerative potential.

Progesterone is a cholesterol‐derived hormone, which plays critical role in mammalian reproduction, including oocyte maturation, implantation of the embryo and quiescence of uterine muscle during foetal development.^29^, ^30^ Progesterone also has been demonstrated to possess many other functions, such as neuroprotective effect in ischemic/reperfusion and traumatic brain injury by reducing inflammation and attenuating neuronal death.^31^, ^32^, ^33^ Cardiovascular effects of progesterone have also been reported; its anti‐atherosclerotic effect occurs by inhibiting proliferation and migration of aortic smooth muscle cells. Furthermore, progesterone‐mediated cardioprotective effects against bisphenol A‐induced arrhythmogenesis and doxorubicin‐induced apoptotic cell death were also observed.^34^, ^35^ Here, we report that progesterone could promote adult CM proliferation and improve cardiac function after MI, further highlighting the cardioprotective role of progesterone. In this study, we administrated progesterone 48 hours after MI. Since CM cell death mainly occurs within the first day after MI,^36^, ^37^ the progesterone‐mediated improvement in cardiac function and remodelling can be mainly attributed to enhance CM proliferation in peri‐infarct area, instead of attenuation of CM death. Our results also revealed that progesterone’s CM proliferative effect is not different between genders. In support of this notion, we observed that there was no difference in cardiomyocyte proliferation between male and female mice at 20 days postnatal, which can be explained by the phenomenon that the progesterone peak at around 20 days postnatal exists in both male and female due to adrenal secretion, and there is no difference for the plasma progesterone levels between female and male at 20‐day‐old age.^38^

**Figure 8:**
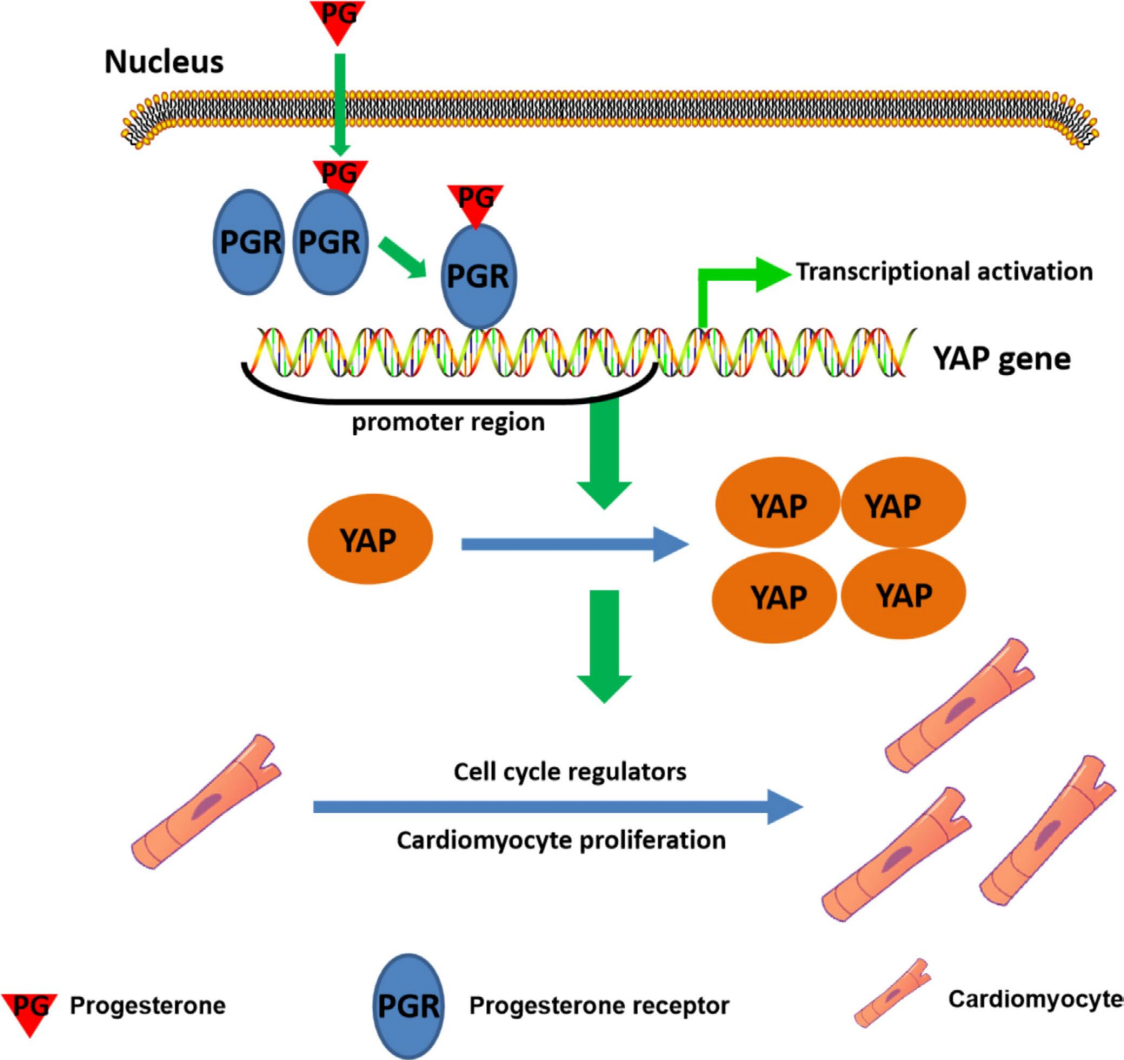
Progesterone promotes CM proliferation by transcriptional activation of YAP. Schematic diagram proposes that progesterone promotes CM proliferation by transcriptional activation of YAP and thereafter enhancing expression of cell cycle genes. CM, cardiomyocyte; YAP, yes‐associated protein

Some molecular mechanisms that are involved in the regulation of CM proliferation have been identified. For example, cardiac expression of Meis1 increases after birth, which coincides with cell cycle arrest of CMs; its deletion is sufficient in extending the postnatal proliferative window of CMs and re‐activation of cardiac regeneration in the adult heart.^39^ Other important regulators of CM proliferation include YAP, GATA4, TBX20 and FSTL1.^21^ Our results show that administration of progesterone promoted YAP expression with the biggest changed fold among these factors, indicating that YAP may be involved in the proliferative effect of progesterone. YAP is one of the downstream effectors of the Hippo pathway, which plays a fundamental role in the regulation of organ growth during development.^40^, ^41^ Mechanistically, YAP acts together with TAZ‐TEAD complexes as transcription factor to bind to gene enhancers and interacts with RNA polymerase II and/or chromatin‐remodelling factors to drive or repress the expression of target genes. Thus, YAP plays important roles in many biological processes, including cell proliferation, migration and fate determination.^42^ Notably, it is well established that activation of YAP by its overexpression or inhibition of Hippo signalling can provoke CM proliferation, thus enhancing heart regeneration and repair after injury, including MI.^41^, ^43^, ^44^, ^45^ Our data show that increased expression of YAP in progesterone‐treated CMs occurred in parallel with the activation of YAP target genes, including ANKRD1, CYR61 and CTGF. Conversely, the pro‐proliferative effect of progesterone could be diminished by YAP silencing. Thus, we can conclude that up‐regulation of YAP is the main mechanism for the progesterone‐mediated promotion of CM proliferation (Figure 8). Furthermore, in accordance with progesterone’s well‐established ability in regulating target gene expression, we showed binding of progesterone receptor in YAP promoter, suggesting progesterone promotes YAP expression via progesterone receptor‐mediated transcription activation of the YAP promoter. Herein, we report for the first time that YAP is a direct target gene of the progesterone receptor in CM. As previously reported,^41^ YAP plays an important role in cardiomyocyte proliferation, suggesting it as a therapeutic target gene in cardiac regeneration. However, it should be noticed that gene therapy technology is still far from clinical application due to low organ specificity and other drawbacks.^46^ Furthermore, sustained activation of YAP may lead to adaptive cardiac hypertrophy upon hypertrophic stress and lead to tumorigenesis in other organs.^47^, ^48^ If using progesterone as a therapy method, the duration and dosage can be well controlled, thus avoiding the side effects resulted from sustained and non‐specific YAP activation.

In summary, the current report unveiled progesterone withdrawal as a new mechanism for postnatal CM cell cycle arrest. In addition, progesterone supplementation promotes both neonatal and adult CM proliferation through up‐regulation of YAP signalling to improve cardiac repair after injury. Our findings may help to advance therapeutics in the field of cardiac regenerative medicine.

## CONFLICT OF INTEREST

The authors declare that they have no competing interest.

## AUTHOR’ CONTRIBUTIONS

Chunyu Zeng and Zaicheng Xu conceived the project and designed the experiments. Cong Lan, Nian Cao, Caiyu Chen, Shuang Qu, Chao Fan, Andi Zeng, Hao Luo, Cheng Yu, Yuanzheng Xue, Hongmei Ren, Liangpeng Li, and Hongyong Wang conducted experiments. Cong Lan wrote the manuscript. Pedro A Jose, Chunyu Zeng and Zaicheng Xu revised the manuscript.

## Supporting information

Figures S1‐S10

## Data Availability

The data that support the findings of this study are available from the corresponding author on reasonable request.
