# Supplementary material for: Progesterone, via yes‐associated protein, promotes cardiomyocyte proliferation and cardiac repair
Source: Cell Prolif. 2020 Oct 12;53(11):e12910. doi: 10.1111/cpr.12910 (PMC7653240; doi:10.1111/cpr.12910)
Supplement: Supplementary file 1 — Figures S1‐S10 [file CPR-53-e12910-s001.doc]

**-Supplementary Materials-**

**Supplementary figures**


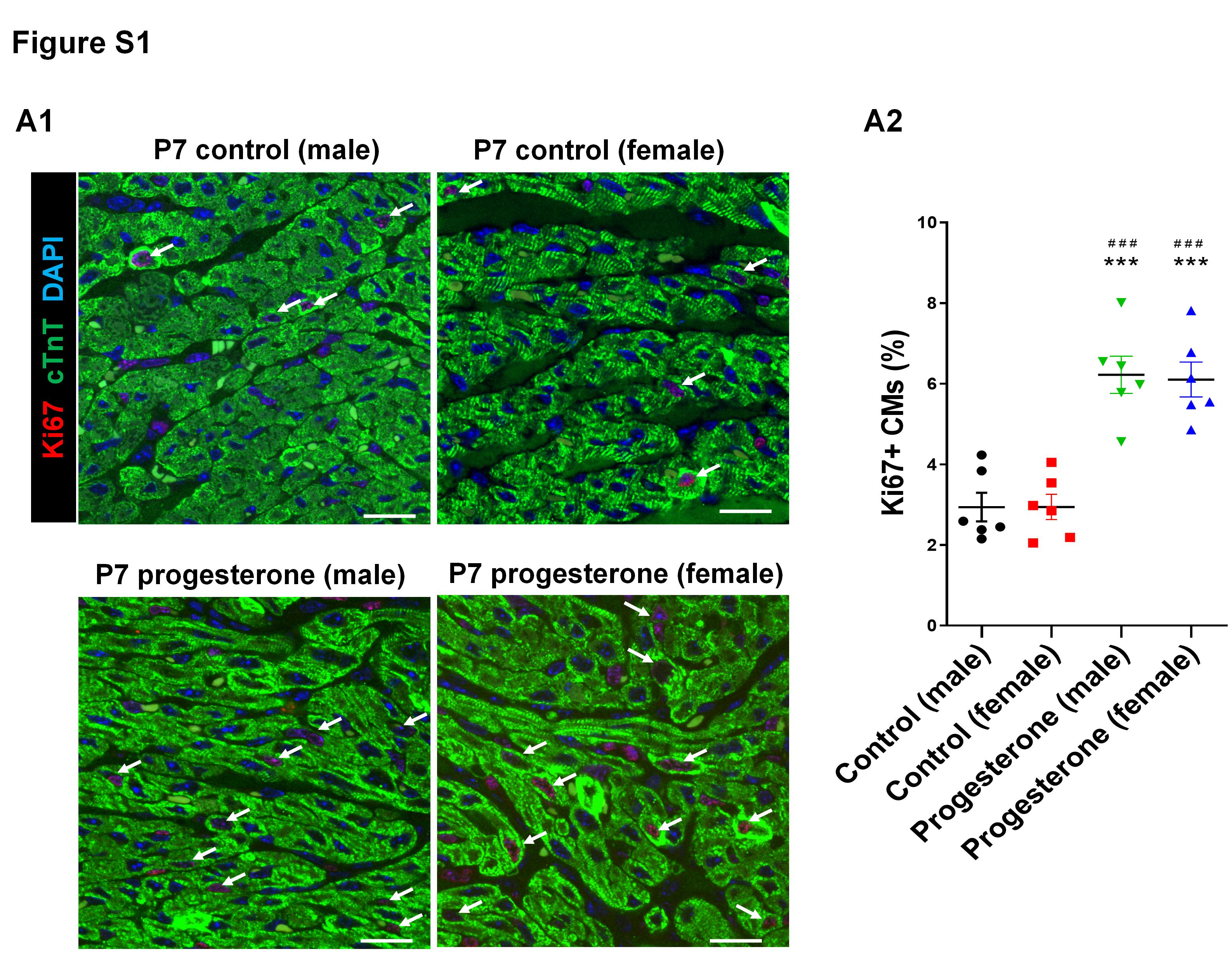


**Figure S1.** Effect of progesterone on cardiomyocyte proliferation was not different between genders. Daily intraperitoneal injection of progesterone (8 mg/kg) or control vehicle (corn oil) in both male and female mice from P1 to P6 was performed and hearts were harvested at P7. Representative images of Ki67 immunostaining and quantification of percentages of Ki67^+^ CMs (n=6) were shown. Scale bars are 20 µm. ^***^P<0.001 vs. Control (male); ^###^P<0.001 vs. Control (female).


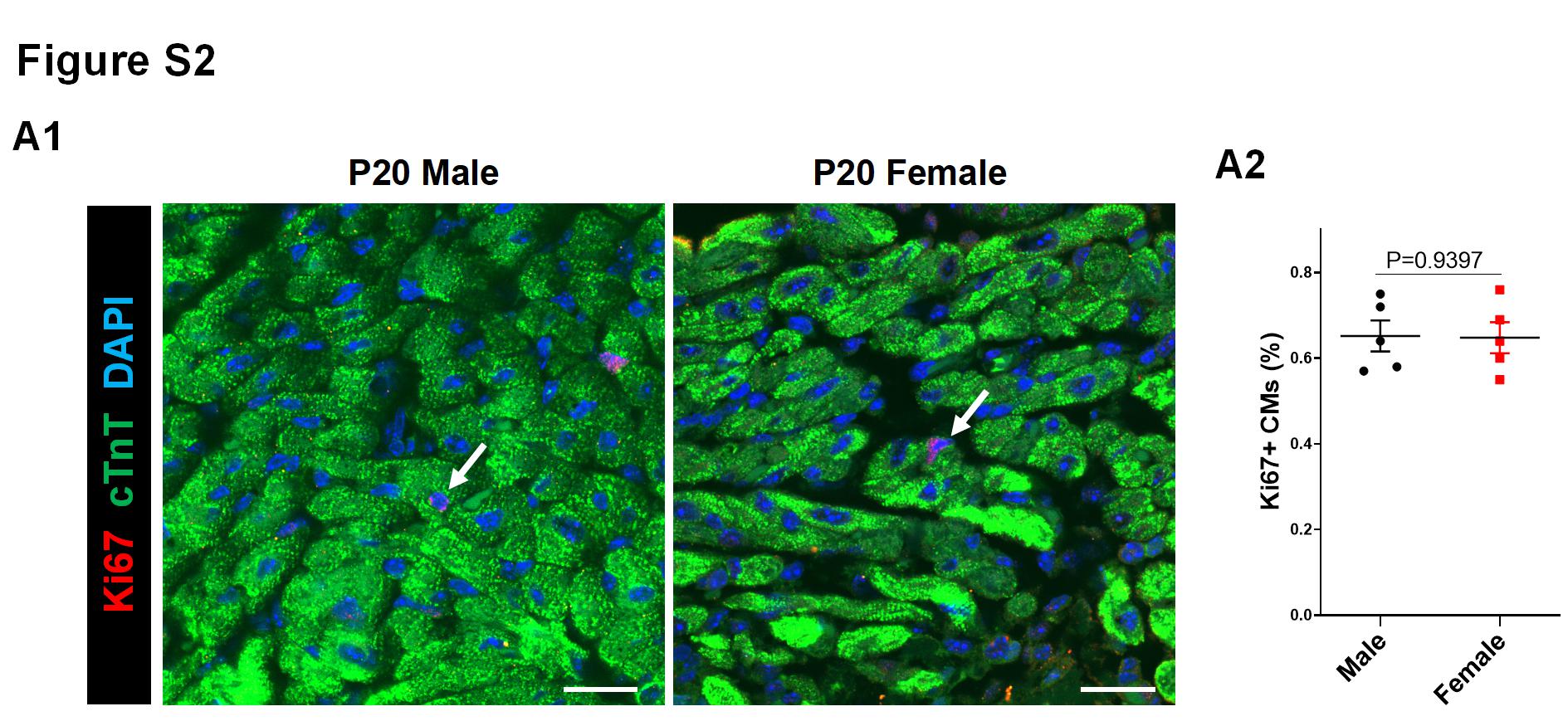


**Figure S2.** Comparation of cardiomyocyte proliferation between male and female mice at postnatal day 20. Hearts of male and female mice at postnatal day 20 (P20) were harvested and cardiomyocyte (CM) proliferation was analyzed using Ki67 immunostaining. Representative images (A1) and quantification (A2) of percentages of Ki67^+^ CMs were shown (n=5). Scale bars are 20 µm.


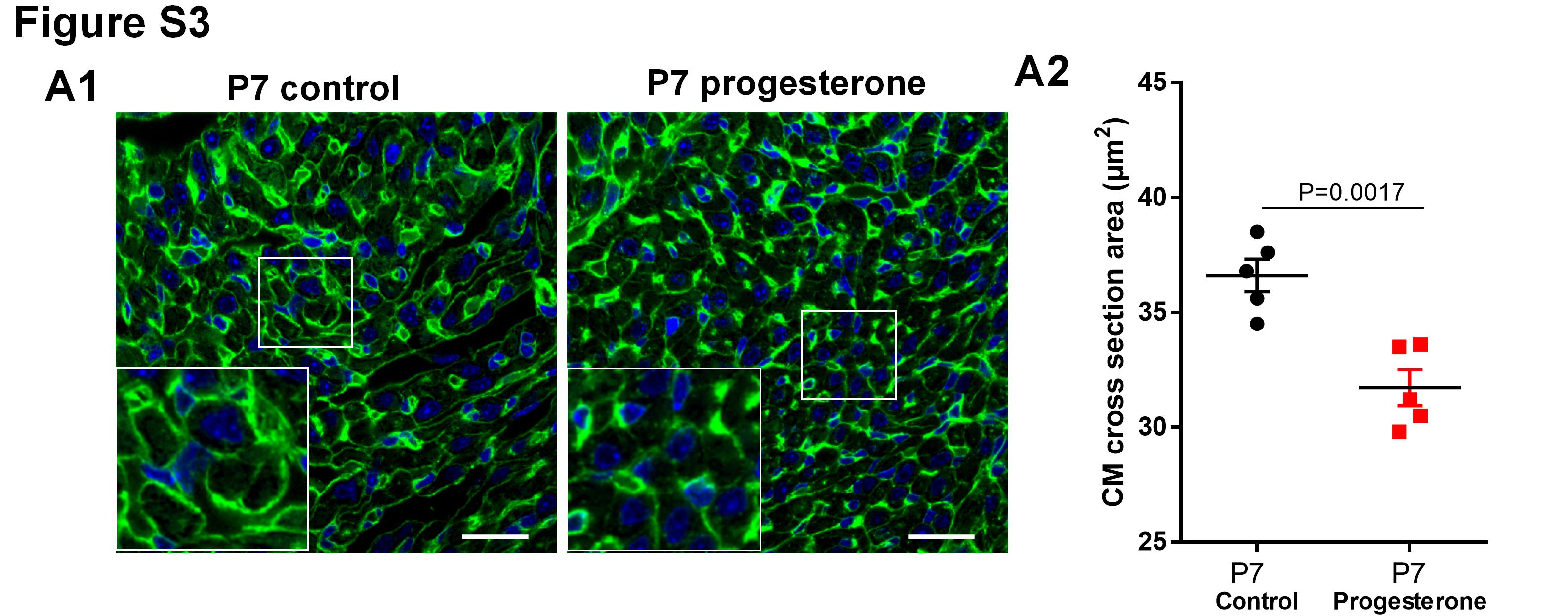


**Figure S3.** Effect of progesterone on CM cross section area. Daily intraperitoneal injection of progesterone (8 mg/kg) or control vehicle (corn oil) in mice from P1 to P6 was performed and hearts were harvested at P7. **A:** Representative images (A1) and quantification (A2) of the cross-section area of CMs measured by WGA staining (green fluorescence). Eighty to one hundred cells were randomly selected per section and 4 sections were measured per heart (n=5). Scale bars are 20 µm.


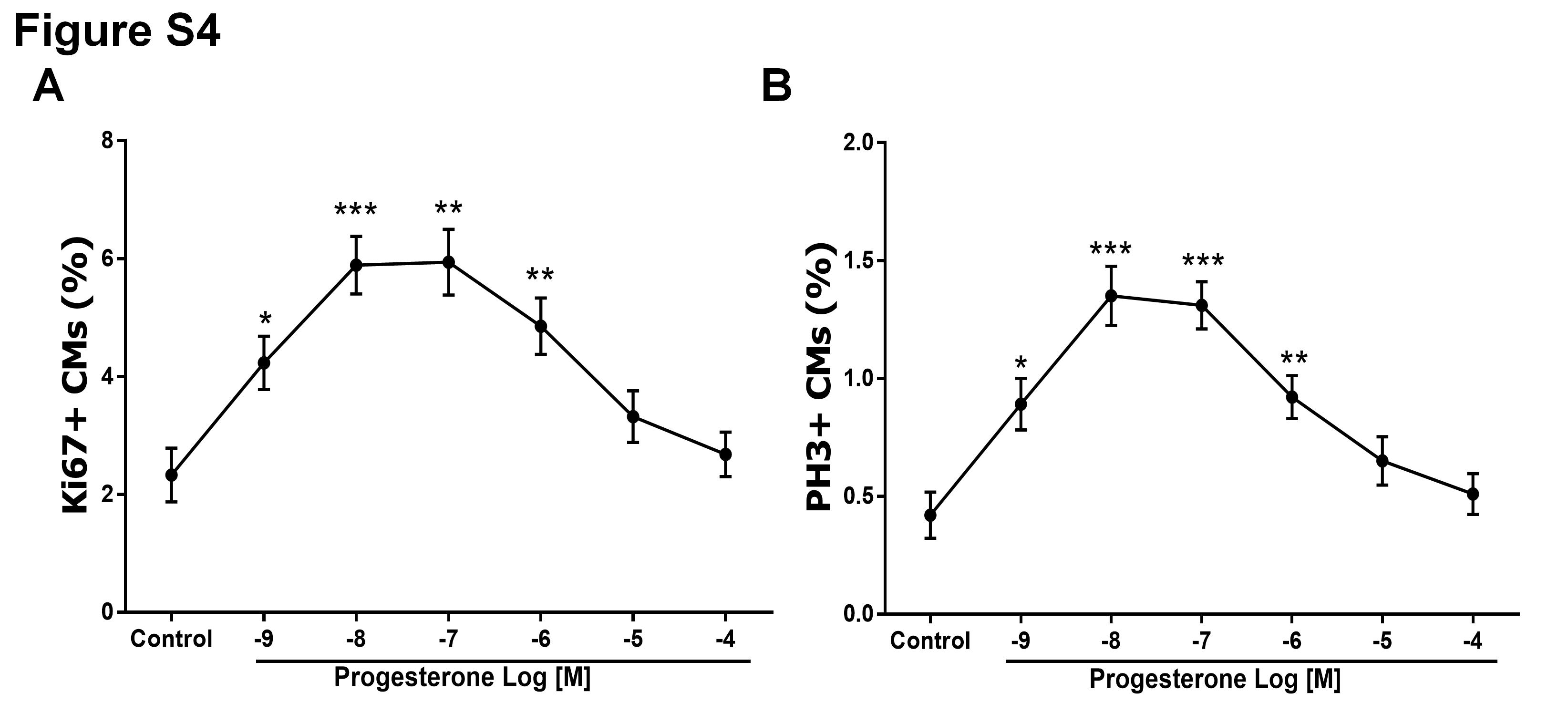


**Figure S4.** Progesterone promotes CM proliferation *in vitro* in a concentration-dependent manner. P7 CMs were treated with control vehicle (DMSO) or various concentrations of progesterone (10^-9^-10^-4^M) for 24 hours. Thereafter, CM proliferation was evaluated by percentage of Ki67^+^ CMs (**A**) and PH3^+^ CMs (**B**). One thousand cells were randomly selected and analyzed for each group and 5 independent experiments were conducted. (^*^P<0.05, ^**^P<0.01, ^***^P<0.001) vs. 0 Mol/L (Control).


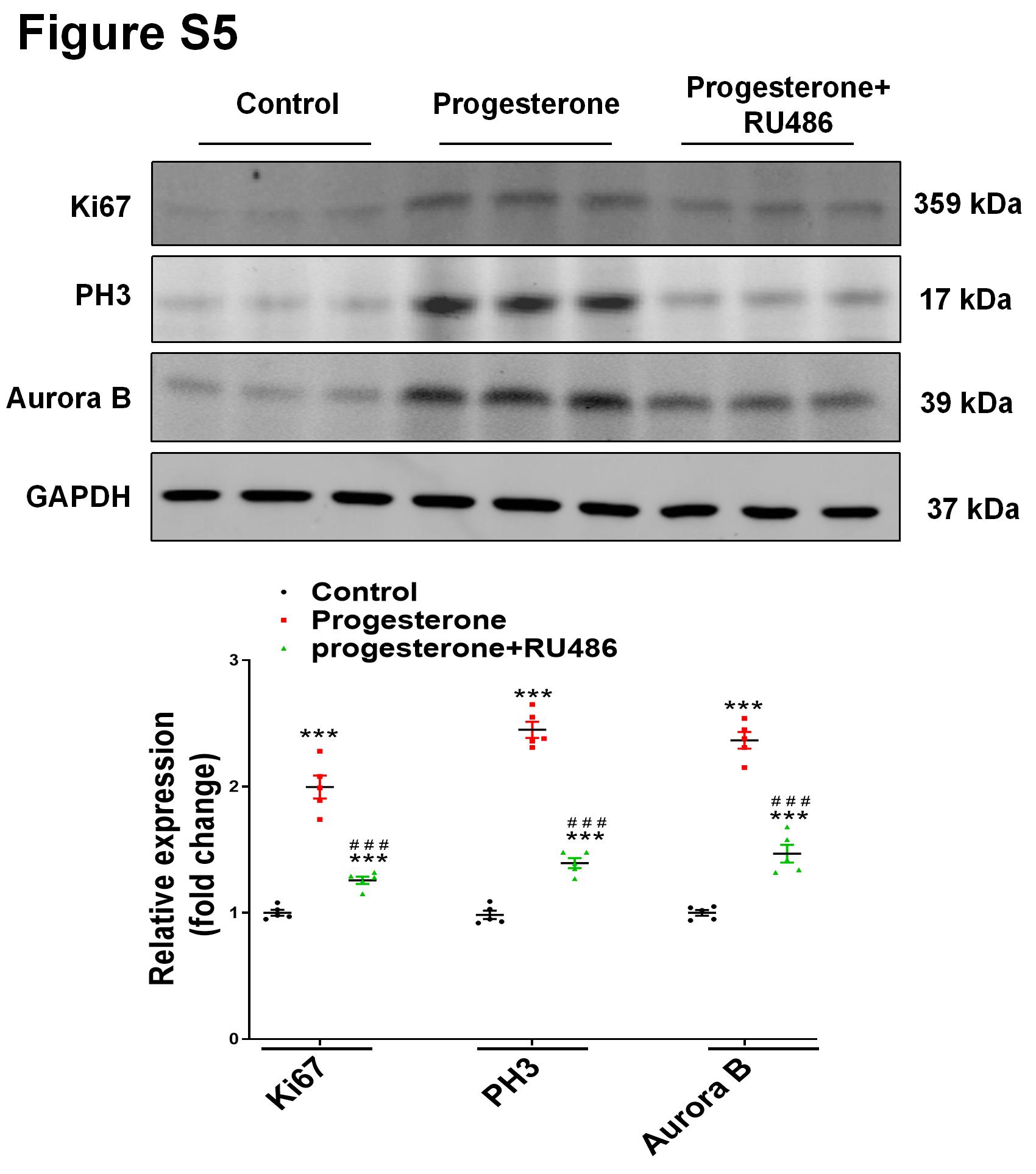


**Figure S5.** Progesterone increases expression of proliferative markers Ki67, PH3 and Aurora B in cultured CM *in vitro* in a progesterone receptor-dependent manner. Cultured P7 CMs were treated with control vehicle or progesterone (10^-7^M), alone or in combination with the progesterone receptor inhibitor RU486 (10^-6^M). Protein levels of proliferation markers were analyzed by Western blot 24 hours after progesterone stimulation, ^**^P<0.01, ^***^P<0.001 vs. Control; ^##^P<0.01, ^###^P<0.001 vs. Progesterone (n=4).


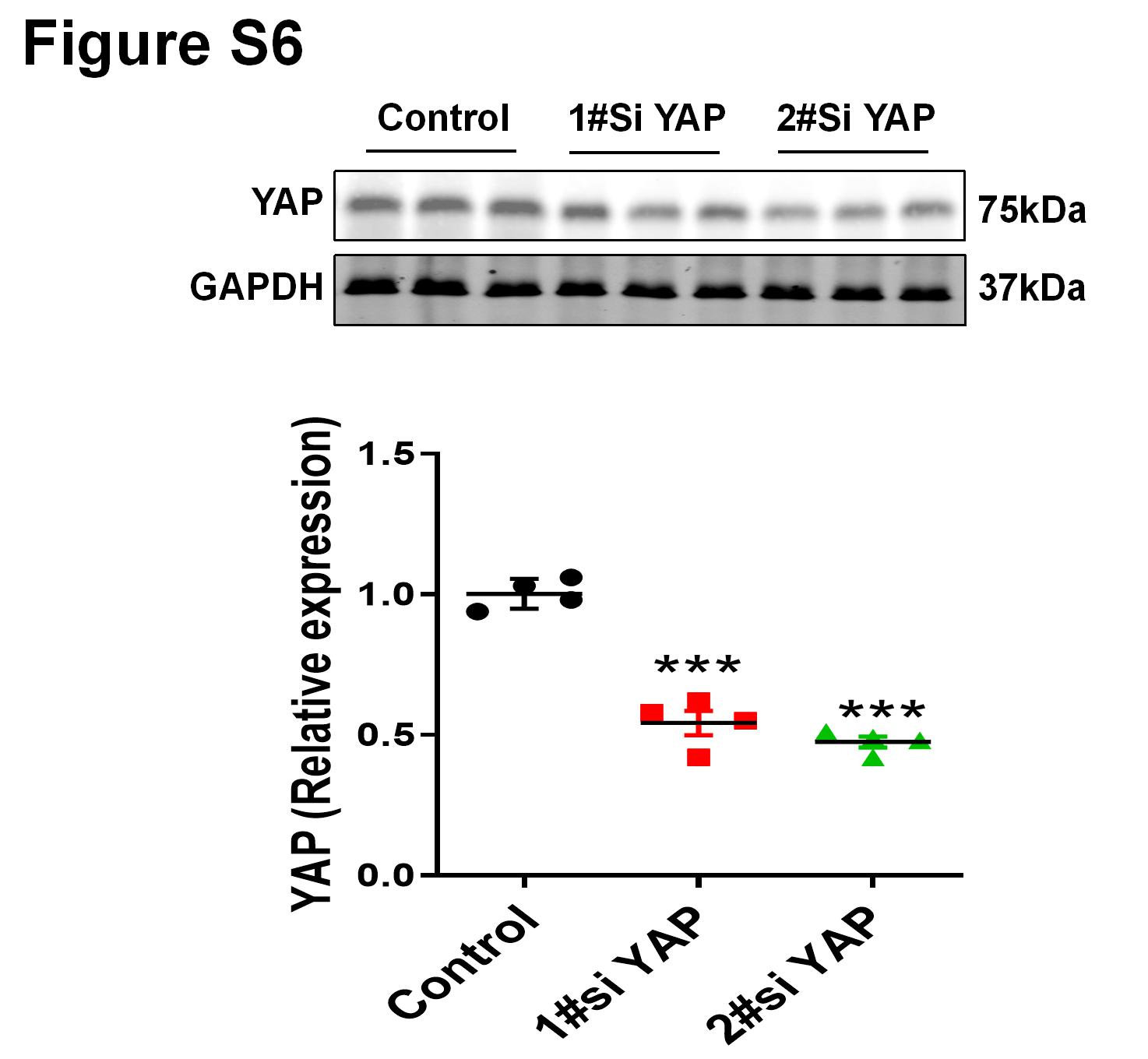


**Figure S6.** Down-regulation of YAP expression by siRNAs in CMs. P7 CMs were transfected with two individual siRNAs against YAP. YAP expression was determined by western blot analysis. ^***^P<0.001 vs. Control (n=4).


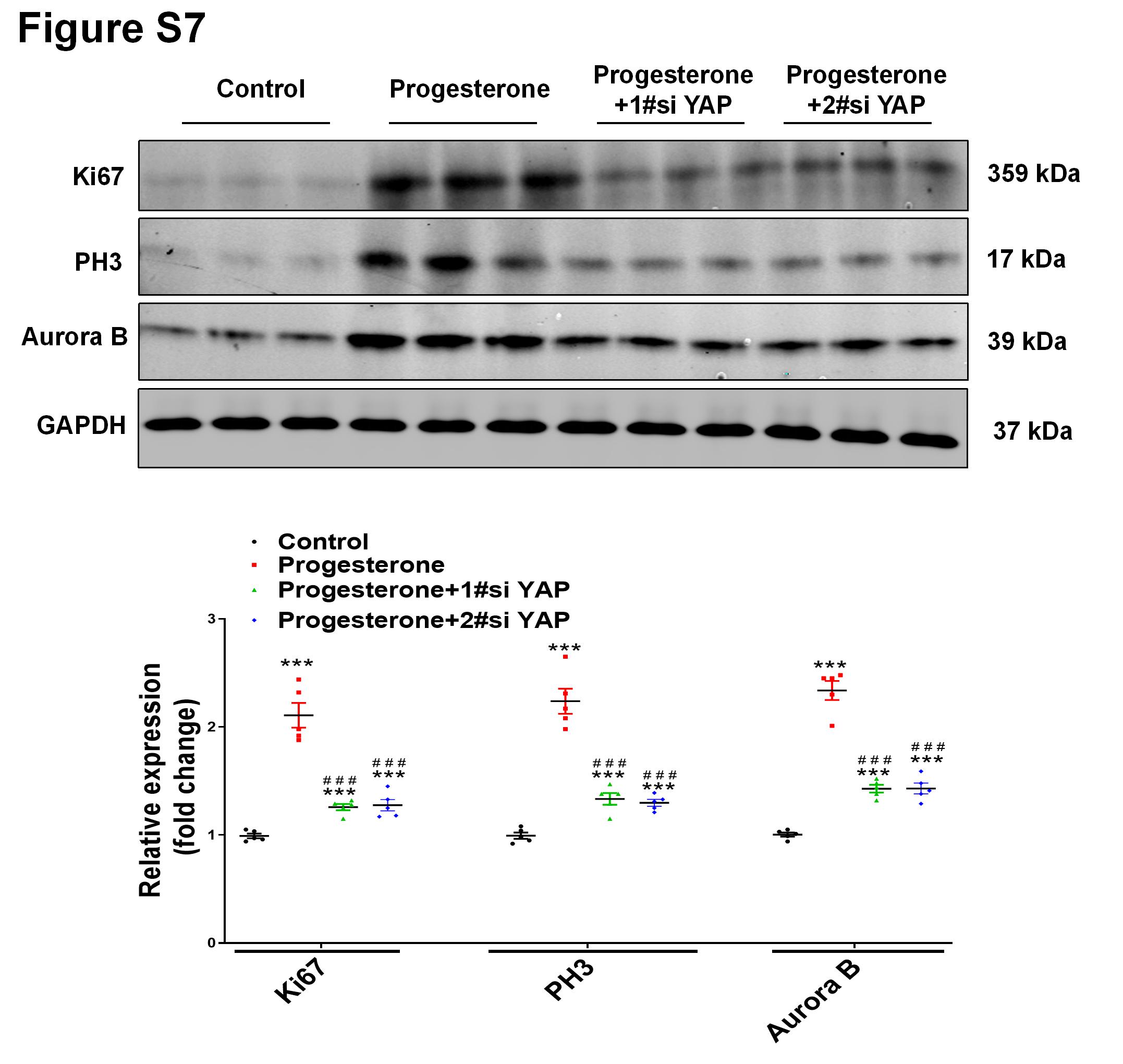


**Figure S7.** Progesterone increases expression of proliferative markers Ki67, PH3 and Aurora B in cultured CM *in vitro* in a YAP dependent manner. Cultured P7 CMs were transfected with two individual YAP siRNAs to knockdown YAP expression and treated with control DMSO or progesterone (10^-7^M) for 24 hours. Protein levels of proliferation markers were analyzed western blot analysis. ^***^P<0.001 vs. Control; ^###^P<0.001 vs. Progesterone (n=4).


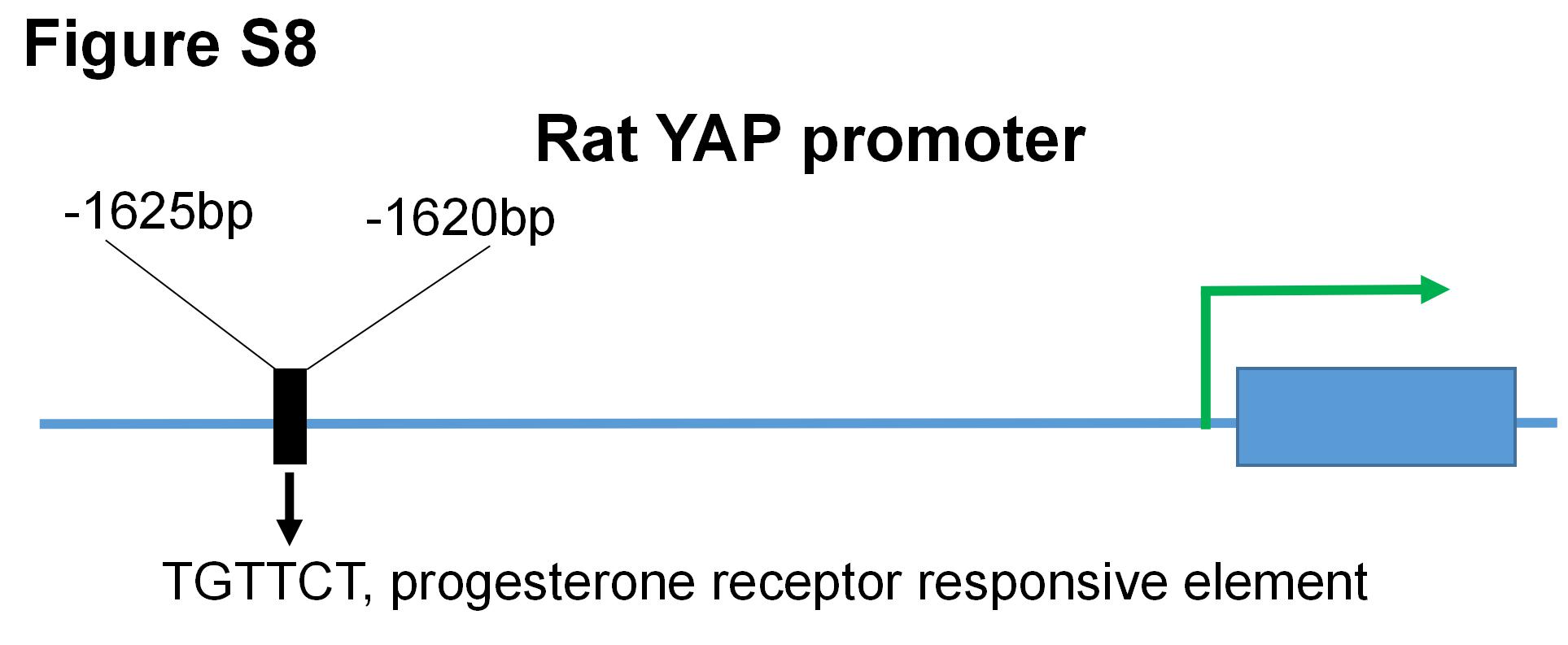


**Figure S8.** Analysis of progesterone receptor binding motif in the rat YAP promoter.


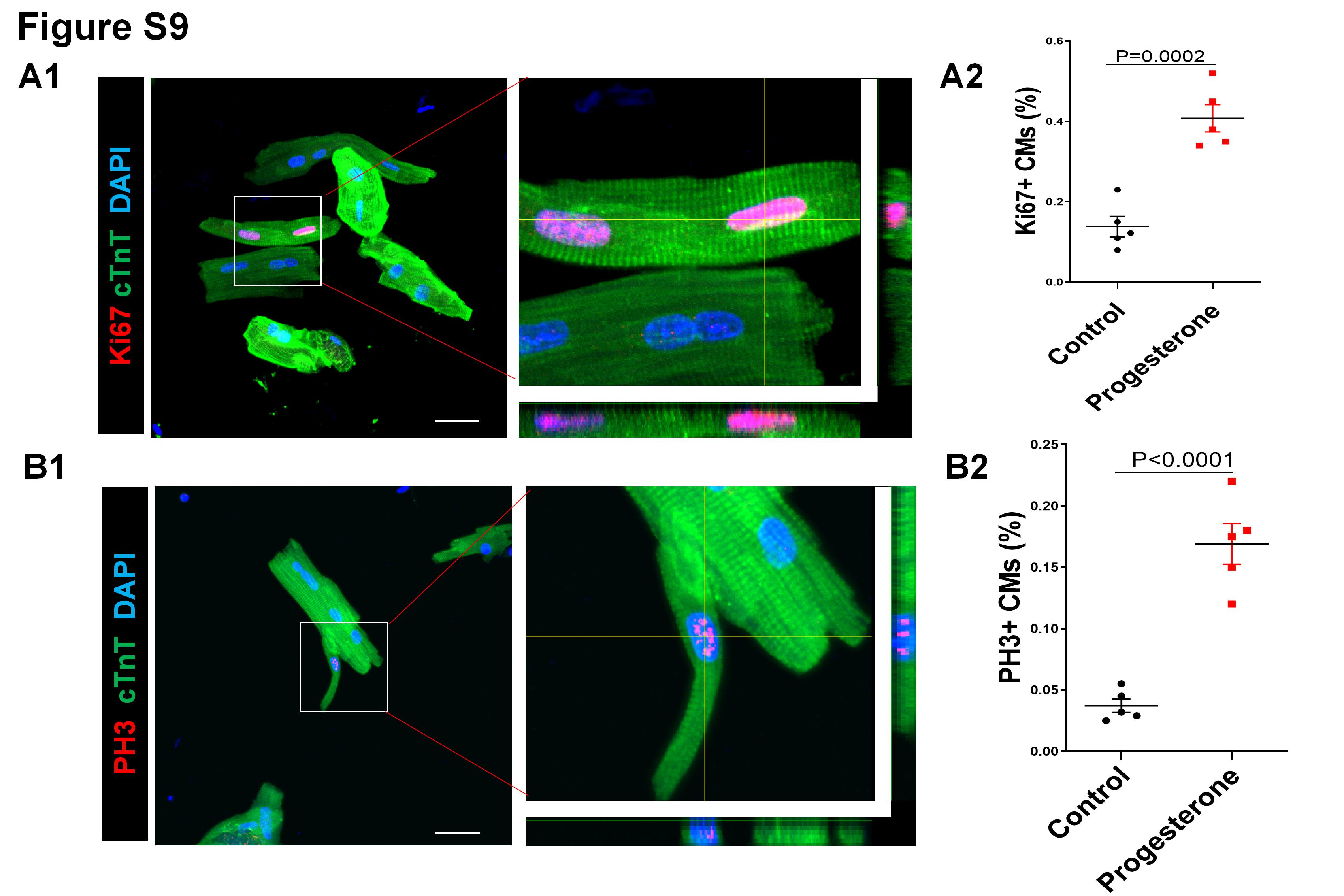


**Figure S9.** Progesterone increases adult CM proliferation after MI. Adult mice were subjected to MI by ligation of the left anterior descending coronary artery and intraperitoneally injected daily with progesterone (8 mg/kg) or control vehicle (corn oil). Hearts were harvested at P7 and digested to isolate the CMs, followed by immunostaining to measure the cell size and count Ki67 and PH3 positive CMs. **A-B:** Representative images with z-stacking (A1-B1) and quantification of percentage of Ki67 (A2) and PH3 (B2) positive CMs are shown (more than 10000 cells were randomly selected in each heart and 5 mice were analyzed in each group). Scale bars are 40 µm.


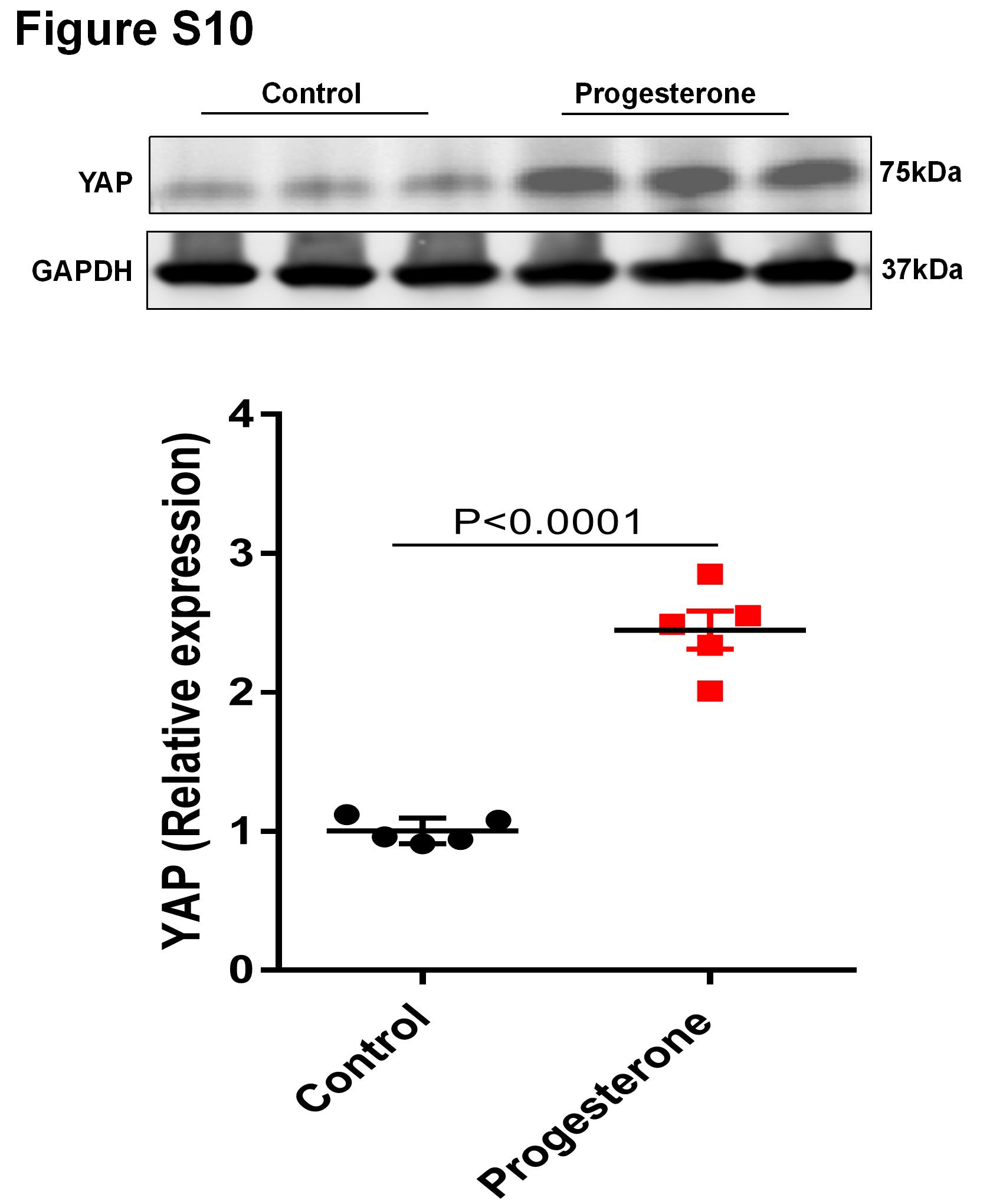


**Figure S10.** Progesterone increases YAP expression in CMs after MI. Adult mice were subjected to MI and intraperitoneally injected daily with progesterone (8 mg/kg) or control vehicle (corn oil). The treated hearts were harvested at day 7 after MI. Then CMs were isolated and subjected to western blot analysis to determine YAP expression (n=5).
